# Adiponectin suppresses tumor growth of nasopharyngeal carcinoma through activating AMPK signaling pathway

**DOI:** 10.1186/s12967-022-03283-0

**Published:** 2022-02-14

**Authors:** Zongmeng Zhang, Jinlin Du, Hui Shi, Shuai Wang, Yunjing Yan, Qihua Xu, Sujin Zhou, Zhenggang Zhao, Yunping Mu, Chaonan Qian, Allan Zijian Zhao, Sumei Cao, Fanghong Li

**Affiliations:** 1 The School of Biomedical and Pharmaceutical Sciences, Guangdong University of Technology, No.100 Waihuanxi Road, Guangzhou Higher Education Mega Center, Guangzhou, 510006 China; 2 Department of Epidemiology and Health Statistics, School of Public Health, Guangdong Medical University, Dongguan, 523808 China; 3 Department of Pathology, Jiangsu Province Hospital of Chinese Medicine, Affiliated Hospital of Nanjing University of Chinese Medicine, Nanjing, 210029 China; 4 State Key Laboratory of Oncology in South China, Collaborative Innovation Center for Cancer Medicine, Sun Yat-Sen University Cancer Center, Guangzhou, 510060 China; 5 Department of Cancer Prevention Research, Sun Yat‐sen University Cancer Center, 651 Dongfeng Road East, Guangzhou, 510060 China

**Keywords:** Nasopharyngeal carcinoma, Adiponectin, AMPK, AdipoRon

## Abstract

**Background:**

Adiponectin is an adipocyte-secreted cytokine that enhances insulin sensitivity and attenuates inflammation. Although circulating adiponectin level is often inversely associated with several malignancies, its role in the development of nasopharyngeal carcinoma (NPC) remains unclear. Here, we investigated the clinical association between circulating adiponectin level and NPC, and examined the impact of adiponectin, as well as the underlying mechanisms, on NPC growth both in vitro and in vivo.

**Methods:**

The association between circulating adiponectin level and the risk of developing NPC was assessed in two different cohorts, including a hospital-based case–control study with 152 cases and 132 controls, and a nested case–control study with 71 cases and 142 controls within a community-based NPC screening cohort. Tumor xenograft model, cell proliferation and cycle assays were applied to confirm the effects of adiponectin on NPC growth in cultured cells and in xenograft models. We also investigated the underlying signaling mechanisms with various specific pharmacological inhibitors and biochemistry analysis.

**Results:**

High adiponectin levels were associated with a monotonic decreased trend of NPC risk among males in both the hospital-based case–control study and a nested case–control study. In vitro, recombinant human full-length adiponectin significantly inhibited NPC cell growth and arrested cell cycle, which were dependent on AMPK signaling pathway. The growth of xenograft of NPC tumor was sharply accelerated in the nude mice carrying genetic adiponectin deficiency. An adiponectin receptor agonist, AdipoRon, displayed strong anti-tumor activity in human xenograft models.

**Conclusions:**

These findings demonstrated for the first time that circulating adiponectin is not only inversely associated with NPC, but also controls the development of NPC via AMPK signaling pathway. Stimulation of adiponectin function may become a novel therapeutic modality for NPC.

**Supplementary Information:**

The online version contains supplementary material available at 10.1186/s12967-022-03283-0.

## Background

Nasopharyngeal carcinoma (NPC) has a high prevalence in southeast Asia, especially in southern China [1, 2]. Although Epstein-Barr virus (EBV) infection is the most well-characterized risk factor [3–5], other potential genetic and environmental factors have also been suggested to contribute to the pathogenesis of this malignancy [6, 7]. Obesity has been suggested by some studies as a risk factor of NPC for decades, but the findings from different groups have been inconsistent [8–10]. Recent published studies have proposed that altered levels of adipose-derived adipokines, such as adiponectin, leptin, and resistin, may have contributed to the development of various malignancies [11, 12].

Adiponectin is an adipocytokine almost exclusively secreted by the adipose tissue [13, 14]. Circulating levels of adiponectin are paradoxically reduced in obesity and diabetes [15]. Mounting evidence has clearly shown its intimate involvement in the regulation of cardiovascular function, glucose/lipid metabolism, and chronic inflammation [16–18]. A series of clinical studies have also revealed that circulating adiponectin is inversely associated with the risk of several malignancies, such as multiple myeloma, prostate, breast, colorectal, and pancreatic cancers [19–22]. In fact, adiponectin elicits anti-proliferative effects in different tumor histocytes in vivo and in vitro, including breast, prostate, hepatocellular, and endometrial carcinomas [23–25].

It is still unclear if adipose tissue, through the secreted adiponectin, plays a role in controlling the development of NPC. Herein, we set out to examine the relationship between blood concentrations of adiponectin and the risk of developing NPC in two cohorts from Guangdong province, including a hospital-based case–control study with 152 cases and 132 controls, and a nested case–control study with 71 cases and 142 controls within a community-based NPC screening cohort. Importantly, we also investigated whether, and by what mechanisms, adiponectin directly regulates the growth of NPC cells.

## Materials and methods

### Animal breeding and subcutaneous transplantation

All animal experimental procedures were approved by the Experimental Animal Academic Ethics Committee of Guangdong Pharmaceutical University (gdpulacspf2017064).

Adiponectin deficient mice were generously provided by Professor Philipp Scherer of the University of Texas Southwestern (Dallas, TX, USA). Male nude mice were purchased from the GemPharmatech (Nanjing, Jiangsu, China), and crossed with APN−/− female mice to generate three genotypes of nude mice: APN+/+, APN+/−, and APN−/−. Mice were kept in the Laboratory Animal Center of Guangdong Pharmaceutical University (Guangzhou, Guangdong, China), and maintained in specific pathogen-free conditions with stationary temperature of 23–25 °C and 12-h light/dark cycles.

1 × 10^6^ CNE-2 or 5-8F cells were resuspended in 100 µL PBS and subcutaneously injected into the right armpit region of five- to six-week-old male nude mice. Tumors were measured using digital Vernier calipers every day, with tumor volume calculated using the formula [sagittal dimension (mm) × cross dimension (mm)]^2^/2 and expressed in cm^3^. All animals were sacrificed, tumor tissues were collected, imaged, and weighed.

For AdipoRon administration, four days after injection NPC cells, the mice were randomly allocated into two groups (Vehicle and AdipoRon groups) of 6 mice per group. In the AdipoRon group, mice were intragastrically administered 50 mg/kg AdipoRon suspended in corn oil every other day. In the Vehicle group, mice were administered solvent alone in corn oil.

### Cell culture and regents

The CNE-2 and S18 cell lines were kindly gifted by Professor Chaonan Qian at SYSUCC. HNE2, 5-8F, C666-1 and 6-10B cells were from the Central South University Advanced Research Center (Changsha, Hunan, China). HNE2, 5-8F and 6-10B cells were cultured in RPMI-1640 medium, CNE-2 and S18 cells were cultured in Dulbecco's modified eagle medium containing 4.5 mg/mL glucose, all supplemented with 10% fetal bovine serum (Gibco, Carlsbad, CA, USA), 100 U/mL penicillin and 100 ug/mL streptomycin (Hyclone, Logan, UT, USA). Cells were maintained in a humidified atmosphere of 5% CO_2_ at 37 °C. The cell line was authenticated via deoxyribonucleic-acid profiling using short tandem repeat analysis.

Recombination human full-length adiponectin was dissolved in deionized water to prepare a working stock solution of approximately 0.5 mg/mL (BioVendor, Brno, Czech Republic). AdipoRon was dissolved in DMSO to prepare a working stock solution of approximately 50 mM (Selleck Chemicals, Houston, TX, USA). Compound C was purchased from MedChem Express (Monmouth Junction, NJ, USA), and was prepared as a stock concentration at 10 mM in DMSO and stored at − 80 °C.

### Cell viability and proliferation assays

Cell viability was measured using cell counting kit-8 (CCK-8) (Sangon Biotech, Shanghai, China). Cells were cultured in 96-well plates, with six duplicate wells in each group, and pre-treated in 100 μL medium with or without different concentrations of inhibitors for 1 h, followed by solvent alone, AdipoRon or APN for the indicated period. After incubation, CCK-8 solution was added to each well followed by a further 2 h incubation under 5% CO_2_ at 37 °C. Absorbance was automatically measured at 450 nm with a microplate reader (Infinite F50, Tecan Group Ltd., Mannedorf, Switzerland). The relative cell viability was calculated as the percentage of untreated cells.

Cell proliferation was measured using plate clone formation and 5-ethynyl-2'-deoxyuridine (EdU) assays. CNE-2 cells were plated in 12-well plates and treated with human recombinant adiponectin or AdipoRon. Then, the culture medium was replaced with fresh medium containing adiponectin every 3 days. After 7 days’ treatment, the medium was removed, and cell colonies were fixed and stained with crystal violet (Sangon Biotech). Images were taken with a digital camera, colonies contained more than 50 cells in each well were counted. The EdU assay were preformed according to manufacturer’s instructions (RiboBio, Guangzhou, Guangdong, China). The EdU-positive rate was calculated as EdU-positive cells/Hoechst-stained cells × 100%. The assays were repeated in triplicate.

### Transient transfection with small interfering RNA

The small interfering RNA (siRNA) oligos against AdipoR1, AdipoR2 and scrambled control siRNA were commercially synthesized by RiboBio (Guangzhou, Guangdong, China), and transfected with riboFECT CP transfection reagent (RiboBio, Guangzhou, Guangdong, China) according to the manufacturer’s protocol. The siRNA duplexes used for this study are listed in Additional file 1: Table S2. Two days after transfection, the cells were subjected to total RNA isolation and viability assays.

### Cell cycle assay

CNE-2 cells were incubated in serum-free medium overnight, and then cells were treated with adiponectin or AdipoRon. Cells were collected, washed, and suspended in cold PBS. Cells were then fixed in 70% cold ethanol at 4 °C overnight. After fixation, the cells were washed with PBS twice, resuspended in 0.2 mL PI/RNase staining buffer (BD Biosciences, San Jose, CA, USA) for 30 min at room temperature. The cell cycle distribution was determined by the DxP Athena flow cytometry system (Cytek Biosciences, Fremont, CA, USA), and the percentages of different phases of cell cycle were determined using ModFit LT 5.0 (Verity Software house, Topsham, ME, USA).

### Cell apoptosis assays

PE Annexin V apoptosis detection kit (BD Biosciences, #559763) was used to determine cell apoptosis. Cells treated with the indicated drug concentrations. After treatment, we harvested the cells, washed them twice with PBS, and stained them using Annexin V-PE and 7-AAD for 15 min in the dark, followed by analysis using the DxP Athena flow cytometry system (Cytek Biosciences). The upper right quadrant represents late apoptotic cells, and the lower right quadrant represents early apoptotic cells. The assessment of the apoptosis rate was the sum of early and late apoptosis.

### RNA extraction and qRT-PCR

Total RNA was extracted from cell by using Trizol reagent (Sigma; T9424). The quantity and quality of RNA were determined using a ScanDrop2 nano-volume spectrophotometer (Analytik Jena), and reversely transcribed based on the HiScript II Q RT kit (Vazyme; R223) according to the manufacturer’s instructions. Amplification and real-time detection were performed on a qTOWER3 G real-time PCR system (Analytik Jena) by using ChamQ Universal SYBR qPCR Master Mix (Vazyme; Q711) in 20 μL reaction. The relative expression levels of each targeted gene were normalized by subtracting the corresponding mouse β-actin threshold cycle (CT) values by using the ΔΔCT comparative method. Three biological replicates per group were used for qPCR. Primers were synthesized by Sangon Biotech (Shanghai, China). Sequences of all primers used are provided in Additional file 1: Table S3.

### Immunoblotting analysis

Cells were collected and homogenized in RIPA lysis buffer containing a protease inhibitor (Beyotime Biotechnology, Shanghai, China). The protein concentration was determined using bicinchoninic acid protein assay kit (Thermo Fisher Scientific, Waltham, MA, USA). Then the equivalent proteins were separated by SDS-PAGE, and transferred on Immobilon-p Transfer Membrane (Millipore, Billerica, MA, USA) with the wet electrical transfer method using Mini Trans-Blot (Bio-Rad Laboratories, Hercules, CA, USA). The membranes were blocked with 5% nonfat dried milk in TBS containing 0.1% Tween-20 for 1 h at room temperature; followed by the primary antibody incubation overnight at 4 °C and the secondary antibody for 1 h at room temperature. The bands were detected with ECL detection system according to the manufacturer’s protocol (Thermo Fisher Scientific) using ChemiDoc XRS + system (Bio-Rad). The gray intensities of bands were measured using ImageJ software (National Institutes of Health, Bethesda, MD, USA) and were normalized for β-actin. The antibodies used were as follows: mouse anti-β-actin (A5316) and mouse anti-GAPDH (G8795) (Sigma-Aldrich, St. Louis, MO, USA); rabbit anti-p21 (#2947), rabbit anti-p27 (#3686), rabbit anti-CDK2 (#2546), rabbit anti-CDK4 (#12790), rabbit anti-Cyclin B1 (#12231), rabbit anti-Cyclin D1 (#2978), rabbit anti-ERK1/2 (#4695), rabbit anti-p-ERK1/2 (#4370), rabbit anti-LKB1 (#3047), rabbit anti-p-LKB1 (#3482), rabbit anti-AMPKα (#5831), and rabbit anti-p-AMPKα (#2535) (Cell Signaling Technology, Danvers, MA, USA); mouse anti-AdipoR2 (sc-514045) (Santa Cruz Biotechnology, Santa Cruz, CA, USA); rabbit anti-AdipoR1 (ab126611) (Abcam, Cambridge, MA, USA); Goat anti-mouse-HRP and goat anti-rabbit-HRP (Jackson ImmunoResearch, West Grove, PA, USA). The densitometry of the bands was quantified using ImageJ software.

### Immunohistochemistry staining

IHC was carried out as described previously [26]. The sections were deparaffinized, rehydrated and performed antigen retrieval with microwave method in 10 mM citrate buffer. The sections blocked with 3% H_2_O_2_ for 15 min, incubated with 5% normal goat serum in PBST for 1 h at 37 °C. Then sections were incubated with primary antibodies mouse anti-AdipoR2 (Santa Cruz; 1:50), rabbit anti-AdipoR1 (Abcam; 1:100), rabbit anti-Ki-67 (#9027, CST) and rabbit anti-CD31 (#77699, CST) at 4 °C overnight. After washing, followed by horseradish peroxidase-conjugated secondary antibody incubation for 1 h. Sections were incubated with developing solution (diaminobenzidine, DAB) and counterstained with hematoxylin (ZSGB-Bio, Beijing, China). Goat anti-mouse-HRP and goat anti-rabbit-HRP (Jackson ImmunoResearch) were used as the secondary antibodies.

### Bioinformatics analyses of AdipoR1 and AdipoR2 expression

Messenger RNA (mRNA) expression data for 566 head and neck squamous cell carcinoma (HNSC) samples were downloaded from The Cancer Genome Atlas (TCGA) data portal (https://xenabrowser.net/datapages/). According anatomic neoplasm subdivision, including 44 tonsil, 9 oropharynx, 143 oral tongue, 87 oral cavity, 3 lip, 128 larynx, 10 hypopharynx, 7 hard palate, 66 floor of mouth, 22 buccal mucosa, 29 base of tongue and 18 alveolar ridge tumors.

Microarray gene expression profiling data including GSE12452 (10 normal controls and 31 NPC samples) [27], GSE53819 (21 normal controls and 18 NPC samples) [28], GSE61218 (21 normal controls and 18 NPC samples) [29], GSE64634 (4 normal controls and 12 NPC samples) [30], GSE103611 (48 NPC samples) [31], GSE132112 (95 NPC samples) [32], and GSE13597 (3 normal controls and 25 NPC samples) [33]. The RNA-seq data of NPC samples including GSE102349 (113 NPC samples) [34] and GSE68799 (4 normal controls and 42 NPC samples). These data were downloaded from the Gene Expression Omnibus (GEO) database.

### Statistical analysis

Data were expressed as mean ± SD. Statistical analyses were performed using GraphPad Prism 7.0 (GraphPad Software, La Jolla, CA, USA). The statistical significance between groups was assessed by Student’s *t* test or by analysis of variance (ANOVA) with Sidak's multiple comparisons test. A value of *P* < 0.05 was considered statistically significant.

## Results

### Association of circulating adiponectin with the risk of NPC

The baseline characteristics of the two cohorts are presented in Table 1. In both case–control studies, patients with NPC and controls had similar gender and age distributions. In the hospital-based case–control cohort, the median circulating adiponectin was significantly lower in the cases versus the controls (1.76 vs 2.94 μg/mL, *P* = 0.003) (Additional file 1: Table S1). Similarly, the median circulating adiponectin also showed a trend of lower value in the cases (vs. the controls) in the nested case–control cohort (1.62 vs 2.21 μg/mL, *P* = 0.06) (Additional file 1: Table S1). These differences were statistically significant among men in both the hospital-based case–control study (1.72 vs 3.50 μg/mL, *P* = 0.002) and the nested case–control study (1.45 vs 2.09 μg/mL, *P* = 0.021) but not among women (Additional file 1: Table S1).

**Table 1:** Circulating adiponectin levels and risk of nasopharyngeal carcinoma in retrospective and prospective cohorts

	Tertiles of total adiponectin levels, μg/mL^a^			
	< 1.34	1.35–7.07	≥ 7.08	*P*_trend_
Analysis 1: Retrospective follow-up, 2009–2015 (N = 284)^b^				
Total				
No. of cases	52	43	37	
No. of controls	40	55	57	
Model 1^c^	1.0 (ref)	0.60 (0.33–1.06)	0.49 (0.27–0.89)	0.020
Model 2^d^	1.0 (ref)	0.76 (0.39–1.46)	0.67 (0.34–1.32)	0.248
Male				
Model 1^c^	1.0 (ref)	0.62 (0.33–1.16)	0.45 (0.24–0.85)	0.014
Model 3^e^	1.0 (ref)	0.71 (0.35–1.43)	0.53 (0.26–1.08)	0.082
Female				
Model 1^c^	1.0 (ref)	0.46 (0.10–2.18)	1.25 (0.21–7.41)	0.335
Model 3^e^	1.0 (ref)	0.88 (0.19–6.09)	2.41 (0.25–23.03)	0.445
Analysis 2: Prospective follow-up, 2008–2016 (N = 213)^b^				
Total				
No. of cases	28	25	18	
No. of controls	43	45	54	
Model 1^c^	1.0 (ref)	0.84 (0.42–1. 67)	0.48 (0.23–1.01)	0.142
Model 2^d^	1.0 (ref)	1.04 (0.32–3.38)	0.26 (0.08–0.83)	0.023
Male				
Model 1^c^	1.0 (ref)	0.86 (0.38–1.93)	0.28 (0.10–0.80)	0.018
Model 3^e^	1.0 (ref)	0.39 (0.06–2.318	0.11 (0.01–0.70)	0.019
Female				
Model 1^c^	1.0 (ref)	0.92 (0.25–3.34)	0.88 (0.27–2.89)	0.842
Model 3^e^	1.0 (ref)	10.28 (0.59–179.22)	1.13 (0.18–6.88)	0.892

^a^The tertiles were defined based on distribution among all control subjects

^b^ORs and 95% CIs were estimated using unconditional logistic regression models in retrospective cohort, and conditional logistic regression models in prospective cohorts

^c^Model 1 was unadjusted

^d^Model 2 adjusted for gender, age, family history, EBV serology status and smoking

^e^Model 3 adjusted for NPC family history, EBV serology status and smoking

Analyses of the risk of NPC associated with circulating adiponectin level were stratified by gender. Among men, we confirmed there was a strong inverse association with risk of NPC in the hospital-based case–control study (highest vs. lowest tertile: OR = 0.45; 95% CI 0.27–0.89, *P*_trend_ = 0.014) and in the nested case–control study (OR = 0.28; 95% CI 0.10–0.80, *P*_trend_ = 0.018) (Table 1). Based on multivariable models, the significance of association was sustained by adjusting for a set of other risk factors for NPC, such as age, family history, smoking, and EBV antibody levels at least in the nested case–control cohort (OR = 0.11; 95% CI 0.01–0.70, *P*_trend_ = 0.019) (Table 1). Taken together, these data show that high adiponectin levels were associated with a statistically significantly reduced future risk of NPC among men, suggesting that altered adiponectin levels may play a role in the etiology of NPC.

### Adiponectin-deficiency exacerbates the growth of NPC cells in vivo

**Fig. 1:**
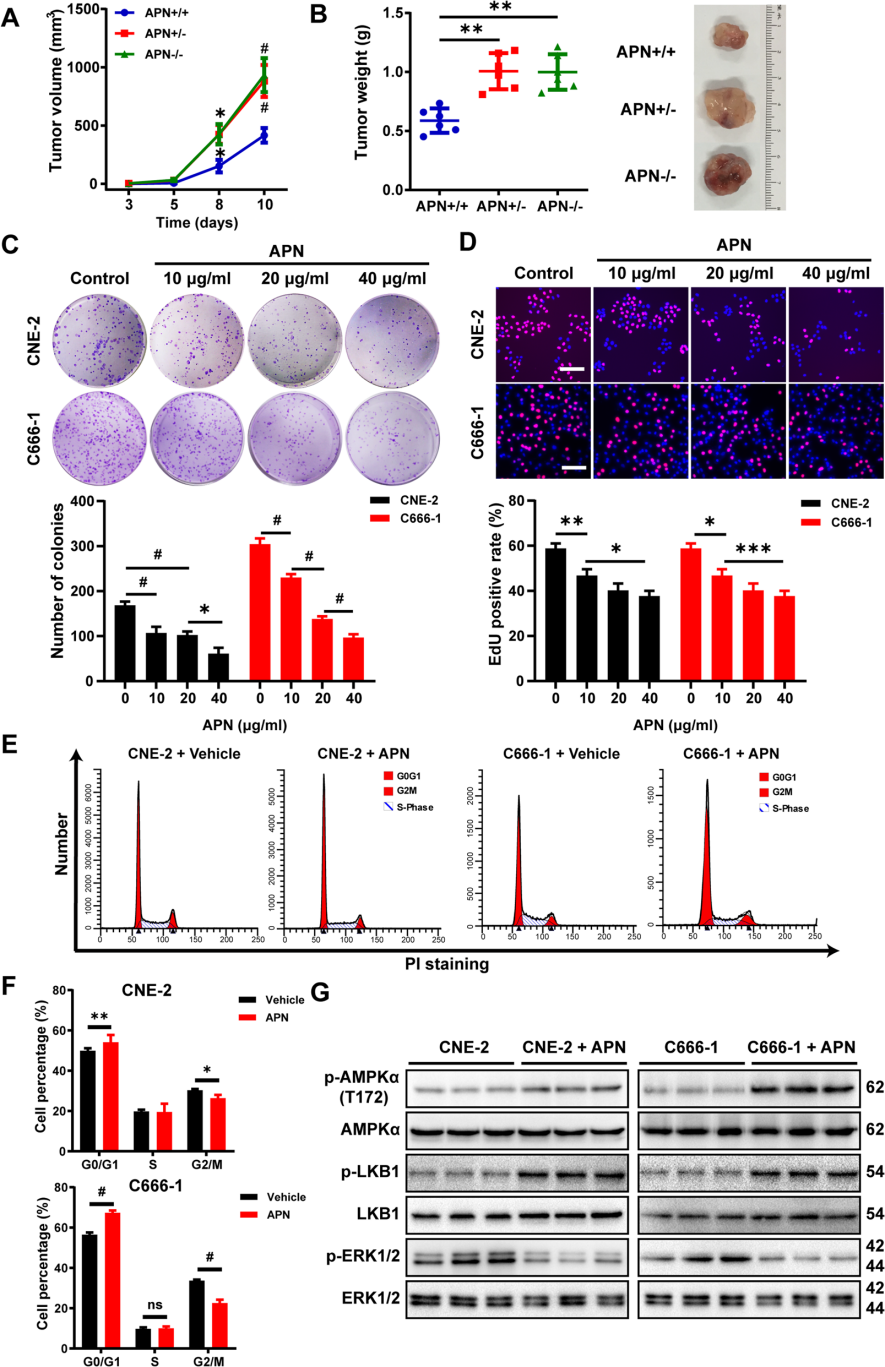
Adiponectin suppresses nasopharyngeal carcinoma growth. **A**, **B** 1 × 10^6^ CNE-2 cells were injected subcutaneously into 5- to 6- week-old adiponectin-deficient nude mice, or the control nude mice (*n* = 6 per group). Tumor growth were monitored by measuring the tumor volume for 10 days. Next, the mice were sacrificed, and tumors were collected, measured, weighed. **C** The plate colony assay was performed to determine colony-formation ability. CNE-2 and C666-1 cells were treated with various concentrations of adiponectin for 7 days. Graphs show the number of colonies. **D** EdU incorporation assay was performed to determine cell proliferation. CNE-2 and C666-1 cells were treated with various concentrations of adiponectin for 48 h. Bars: 50 μm. Graphs show the relative cell proliferation percentage. **E**, **F** CNE-2 and C666-1 cells were incubated with adiponectin (40 μg/mL) for 48 h. At the end of incubation, the cells were collected for FACS analysis. **G** Western blot analysis of p-AMPKα (T172), p-LKB1 and p-ERk1/2 in cultured CNE-2 and C666-1 cells after the treatment with adiponectin (40 μg/mL) for 30 min. Results are presented as mean ± SD of three independent experiments performed in triplicate. *P < 0.05, **P < 0.01, ***P < 0.001, ^#^*P* < 0.0001

The outcomes from these clinical studies warrants further investigation if there exists a causative effect of adiponectin diminution on NPC growth. To this end, we established an NPC tumor model by subcutaneously implanting human NPC (CNE-2) cells-derived xenograft in the immunodeficient nude mice carrying adiponectin deficiency. Compared to the control nude mice, heterozygotic and homozygotic adiponectin deficiency both sharply accelerated tumor growth within even 10 days, and caused significantly increased tumor weight (Fig. 1A, B), indicative of a strong suppressive effect of adiponectin on NPC progression. Consistent with these results in the animal model, co-incubation of recombinant human full-length adiponectin in cultured human CNE-2 and C666-1 cells strongly inhibited the proliferation of cells, as revealed in both the plate clone formation assay and 5-ethynyl-2’-deoxyuridine (EdU)-staining assay (Fig. 1C, D).

The observation that adiponectin suppressed the growth of human NPC-xenograft led us to examine whether adiponectin might modulate cell cycle progression in NPC cells. Flow cytometry analysis showed that co-incubation with adiponectin significantly increased cell population arrested at G_0_/G_1_ stage and decreased the population at G_2_/M stages (Fig. 1E, F). In line with the flow cytometric results, Western blot assays revealed marked reduction of cell cycle regulators, particularly cyclin B1 and cyclin D1, and parallel increase in p21 and p27, following the treatment of CNE-2 and C666-1 cells with adiponectin (Additional file 1: Fig. S1A, B). Such modification of cell cycle regulator appeared to be specific to a sub-group as the levels of some other cell cycle regulatory proteins such as CKD2 and CKD4 were not significantly altered by adiponectin treatment (Additional file 1: Fig. S1A, B). In the same Western blot assay, we also found that adiponectin markedly promoted the activation of AMPK as reflected by the increase of AMPK-phosphorylation (Fig. 1G, Additional file 1: Fig. S1C). LKB1 is known to act as an upstream kinase, directly phosphorylating and activating AMPK [35]. Our results showed that although adiponectin did not affect the level of total LKB1, it increased the level of p-LKB1 (Fig. 1G, Additional file 1: Fig. S1C). Furthermore, adiponectin suppressed the phosphorylation of ERK1/2 (Fig. 1G, Additional file 1: Fig. S1C).

**Fig. 2:**
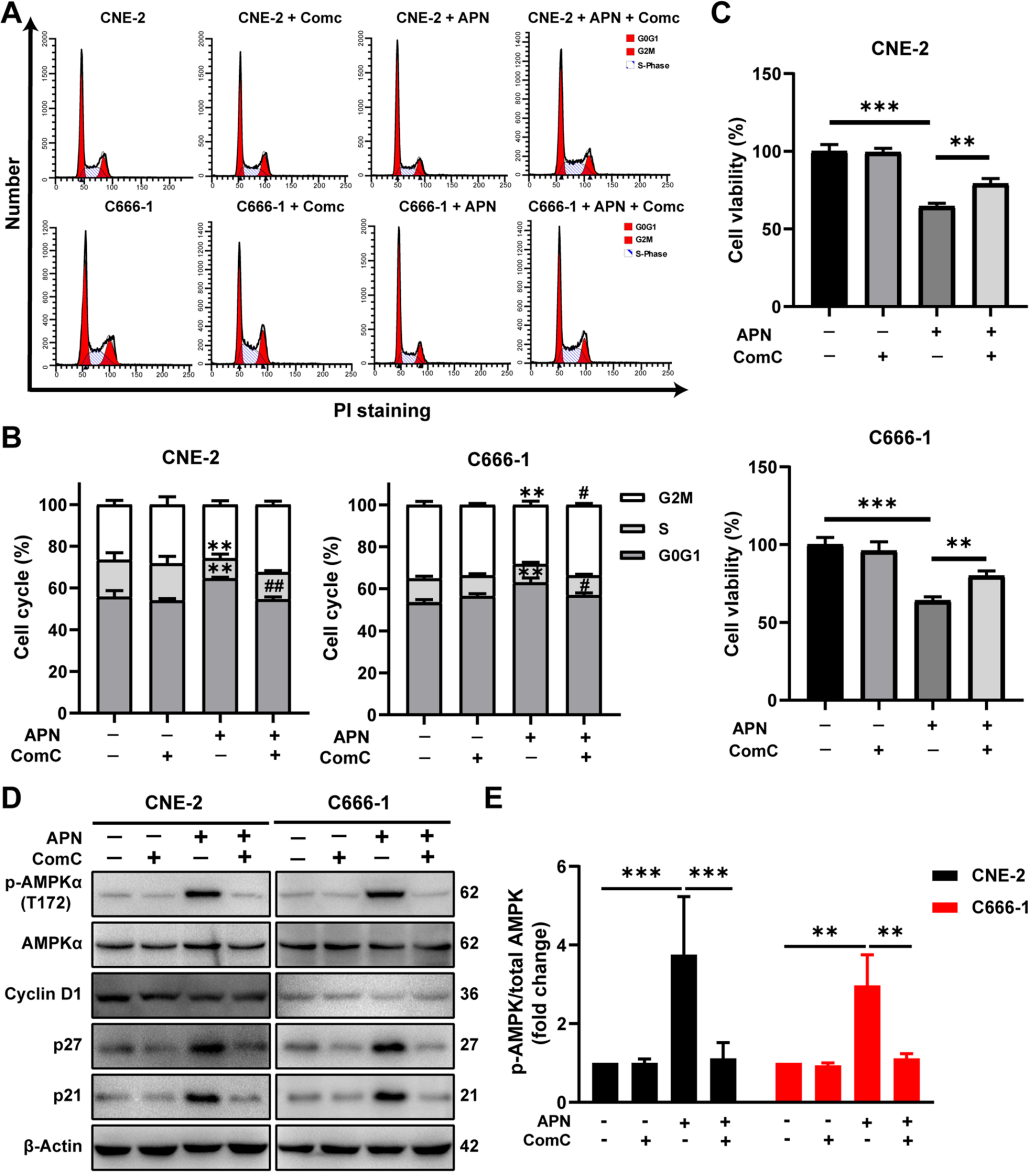
Adiponectin suppresses proliferation of NPC cells via AMPK activation. **A**, **B** CNE-2 and C666-1 cells were pretreated with compound C (10 mM) followed by treatment with adiponectin (40 μg/mL) for 24 h. Cell cycle was then analyzed using flow cytometer. ^#^*P* < 0.05 and ^##^*P* < 0.01 compared to cells treated with adiponectin but not ComC; **P < 0.01 compared with cells treated without adiponectin and ComC. **C** CNE-2 and C666-1 cells viability was determined after treatment with or without adiponectin for 48 h in the presence or absence of ComC (10 μM). **P < 0.01, ***P < 0.001. **D** CNE-2 and C666-1 cells were pretreated with compound C (10 mM), p-AMPKα (Thr172) protein level was then determined by Western blot analysis after the treatment with adiponectin (40 μg/mL) for 30 min; cyclin D1, p21, and p27 protein level was then determined by Western blot analysis after the cells were exposed to adiponectin (40 μg/mL) for 48 h. **E** Quantitative analysis of p-AMPKα level was performed by densitometric analysis. Results are presented as mean ± SD of three independent experiments performed in triplicate. **P < 0.01, ***P < 0.001

To test whether the inhibitory effect of adiponectin on cell cycle progression in NPC cells is dependent on AMPK activation, NPC cells were incubated with adiponectin in the absence or presence of Compound C (ComC), a specific AMPK inhibitor. ComC treatment completely released the NPC cells from the arrest at the G0/G1 phase (Fig. 2A, B). Consistent with such observation, ComC treatment also essentially neutralized the anti-proliferative effect of adiponectin on CNE-2 and C666-1 cells (Fig. 2C). Blockade of AMPK signaling with ComC also neutralized adiponectin-dependent reduction of cyclin D1 as well as the induction of CKIs (p21 and p27) (Fig. 2D, E, Additional file 1: Fig. S2). Collectively, these results demonstrated that adiponectin can directly inhibit the proliferation of human NPC cells by regulating cell cycle-regulatory proteins via activation of AMPK.

### Inhibition of NPC growth by an adiponectin receptor agonist

**Fig. 3:**
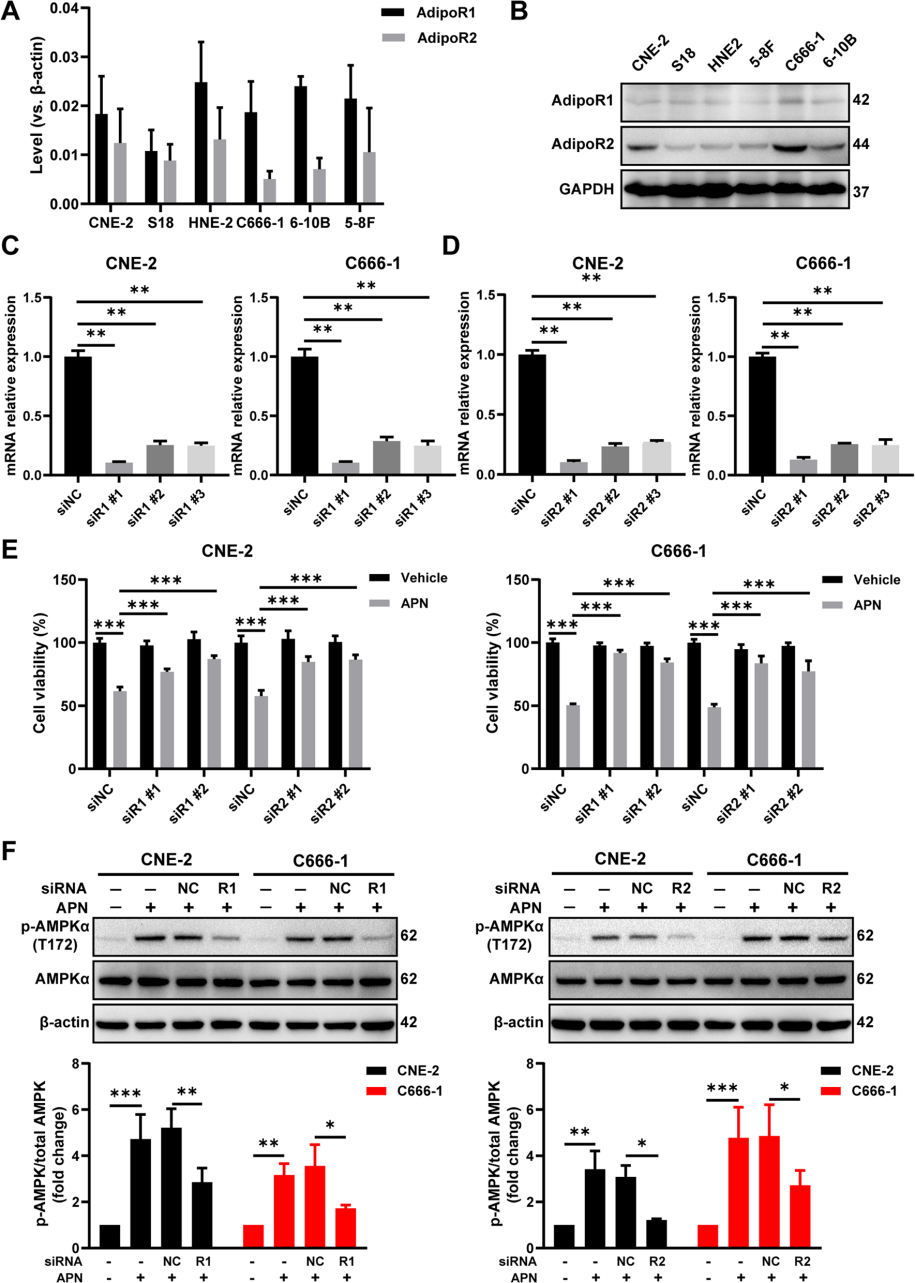
AdipoR1 and AdipoR2 mediate the anti-proliferative effect of adiponectin in NPC cells. **A**, **B** Expression of AdipoR1 and AdipoR2 was determined by qRT-PCR and Western blot in NPC cell lines. **C**, **D** CNE-2 and C666-1 cells were transfected with 50 μM siRNAs of NC, AdipoR1, or AdipoR2. The relative amounts of each AdipoR1/R2 mRNA against β-actin were measured with qRT-PCR. **E** Effect of the knockdown of AdipoR1 and AdipoR2 expression on the cell viability of CNE-2 and C666-1 cells treated with or without 40 μg/mL adiponectin for 48 h. **F** CNE-2 and C666-1 cells were transfected with AdipoR1, AdipoR2 siRNA or NC siRNA and treated with 40 μg/mL adiponectin for 30 min. AMPKα and p-AMPKα (T172) protein levels were determined by Western blot analysis. Quantitative analysis of p-AMPKα level was performed by densitometric analysis and shown in the below part. Results are presented as mean ± SD of three independent experiments performed in triplicate. **P < 0.01, ***P < 0.001

To examine whether the anti-proliferative effect of adiponectin is mediated by adiponectin receptors (AdipoRs) in the human NPC cells, we examined cell lines expressed AdipoR1 and AdipoR2 (Fig. 3A, B). Following reduction of AdipoR1 or AdipoR2 expression via small interfering RNA (siRNA) (Fig. 3C, D) blocked the inhibitory effect of adiponectin on proliferation (Fig. 3E). Under the same condition, adiponectin-induced AMPK phosphorylation was also diminished by the knockdown of AdipoR1 or AdipoR2 expression in NPC cells (Fig. 3F).

**Fig. 4:**
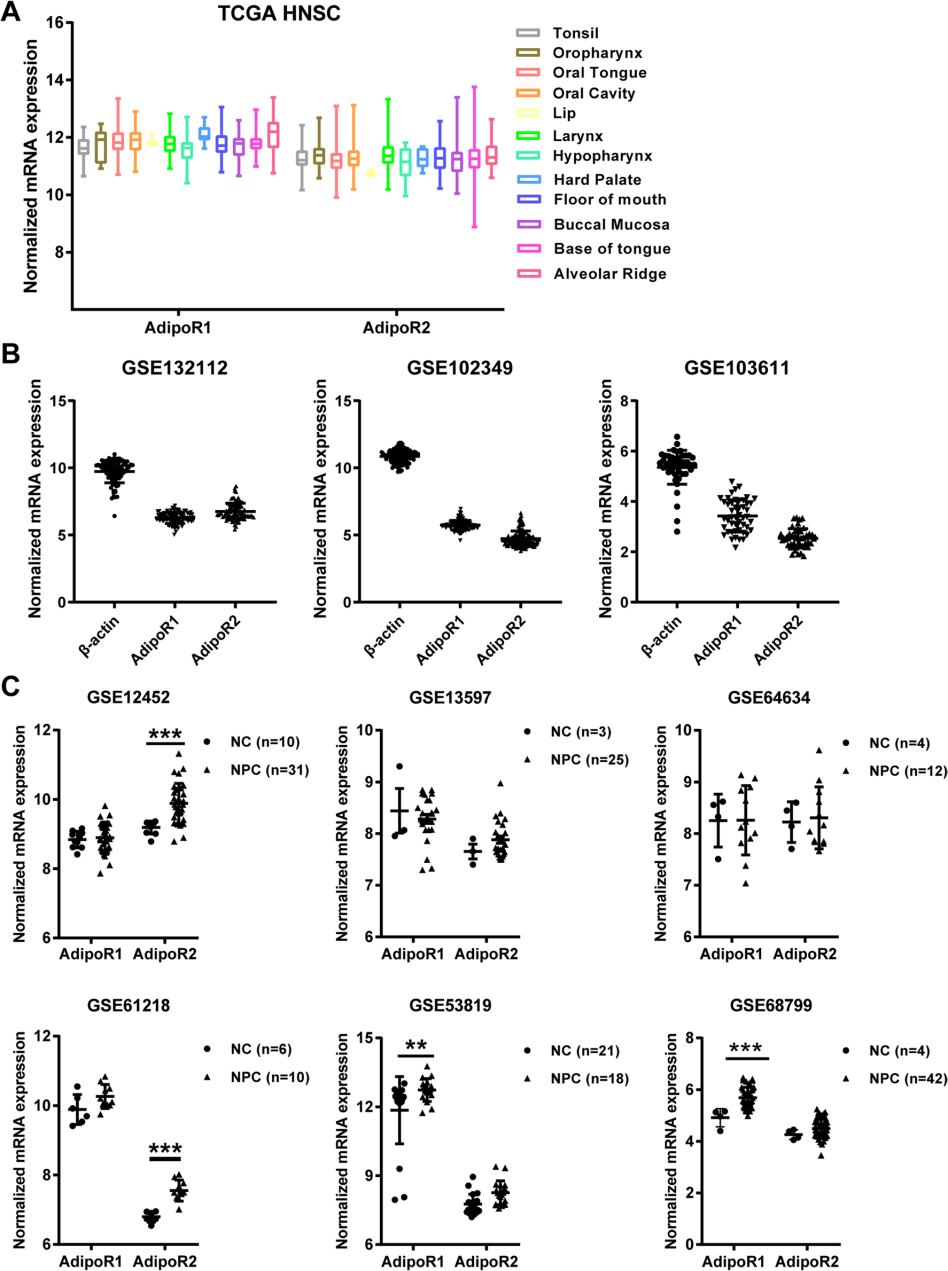
The expression of AdipoR1 and AdipoR2 in human HNSC and NPC. **A** Box plots (derived from TCGA RNA-sequencing dataset) showing the expression of AdipoR1 and AdipoR2 in head and neck squamous cell carcinoma (HNSC). The boxes represent the 25th and 75th percentiles, the lines represent the median, and whiskers show the minimum and maximum points. **B** The mRNA expression levels of AdipoR1 and AdipoR2 were analyzed in NPC tissues from the GEO datasets. **C** The relative mRNA expression of AdipoR1 and AdipoR2 in normal and NPC samples from GEO datasets. Data are expressed as normalized expression units. Data are presented as mean ± SD. **P < 0.01, ***P < 0.001

We further determined if boosting the activity of both adiponectin receptors, AdipoR1 and AdipoR2, will stall the growth of nasopharyngeal carcinoma, if so, what is the impact of altering AdipoRs signaling on cell cycle progression of NPC cells. Initial analysis genomic data in The Cancer Genome Atlas (TCGA) and Gene Expression Omnibus (GEO) found the expression of both AdipoR1and AdipoR2 in various types of head & neck squamous cancers (HNSC) as well as NPC tissues (Fig. 4A, B). Interestingly, further analysis showed that both receptors displayed higher expression levels in NPC than those in the normal nasopharyngeal epithelium (Fig. 4C), which might reflect a compensatory elevation in response to the reduced circulating level of adiponectin in NPC patients. These bioinformatic findings prompted us to explore if a specific adiponectin receptor agonist, AdipoRon, could inhibit the proliferation of human NPC cells.

**Fig. 5:**
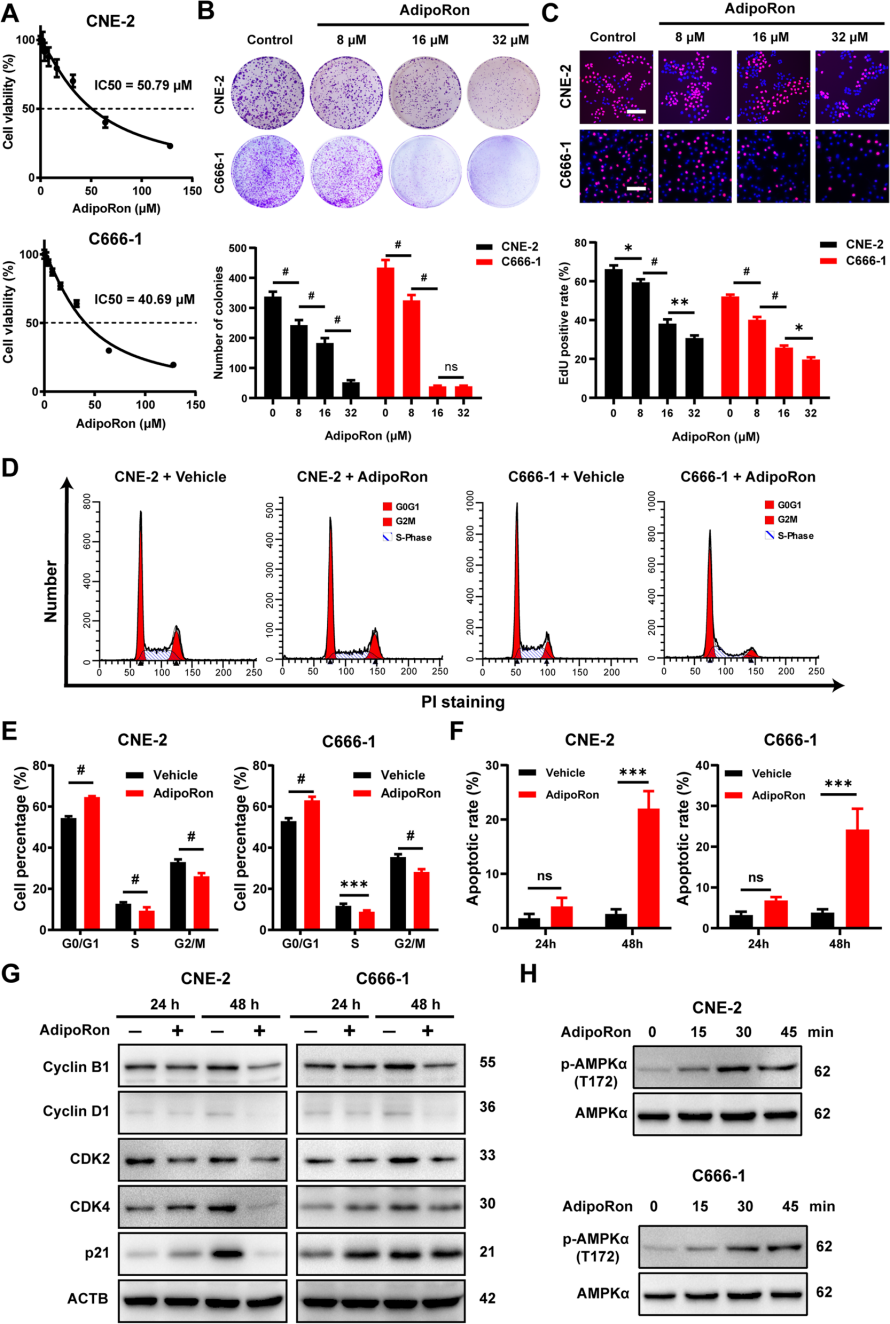
AdipoRon suppresses proliferation of NPC cells in vitro. **A** Cell viability results for CNE-2 and C666-1 cells following the treatment with various concentrations of AdipoRon for 72 h. **B** Colony-formation ability for CNE2 and C666-1 cells treated with vehicle, 16 or 32 μM AdipoRon for 7 days. The graphs show the number of colonies. **C** Cell proliferation for CNE-2 and C666-1 treated with vehicle alone, 16 or 32 μM AdipoRon for 48 h. Bars: 50 μm. Graphs show the relative cell proliferation percentage. **D**, **E** Flow cytometric analysis of cell cycle progression of CNE-2 and C666-1 cells following the treatment of AdipoRon for 24 h. The comparison of the percentage of cells in different cell-cycle phases between AdipoRon-treated cells and corresponding control cells. **F** Annexin V/7-AAD staining of CNE2 and C666-1 cells following 24 h or 48 h of exposure to 50 μM AdipoRon. Cell death was then analyzed using flow cytometer. **G** The cells were treated with vehicle alone or 50 μM AdipoRon for 24 h and 48 h. Western blot analysis was performed to determine the cyclin B1, cyclin D1, CDK2, CKD4 and p21 protein level. **H** CNE-2 and C666-1 cells were treated with 50 μM AdipoRon for the indicated time period. The p-AMPKα (T172) protein levels were determined by Western blot analysis. Results are presented as mean ± SD of three independent experiments performed in triplicate. *P < 0.05, **P < 0.01, ***P < 0.001, ^#^*P* < 0.0001

In NPC cells, AdipoRon treatment of CNE-2 and C666-1 cells generated dose-dependent suppression of cell viability with an IC_50_ at 50.79 μM and 40.69 μM, respectively (Fig. 5A). Even at the doses substantially below IC_50_, AdipoRon still strongly inhibited the proliferation of NPC cells as evident in both the plate clone formation assay (Fig. 5B) and the EdU-staining assay (Fig. 5C).

Flow cytometric analysis showed that co-incubation with AdipoRon significantly elevated cellular population arrested at G0/G1 phase after 24 h treatment (Fig. 5D, E), while inducing apoptosis in the NPC cells at 48 h post treatment (Additional file 1: Fig. S3 Fig. 5F). In complete agreement with the results seen in adiponectin-treated NPC cells, AdipoRon induced significant reduction in the expression of cell cycle regulators, particularly cyclin B1 and cyclin D1, and a parallel increase in p21 (Fig. 5G, Additional file 1: Fig. S4A). Although the levels of CDK2 and CDK4 was not altered in the 24 h treatment duration, their levels were significantly decreased after 48 h (Fig. 5G, Additional file 1: Fig. S4A). In the analysis of these cellular samples, AdipoRon treatment also elicited strong AMPK signaling (Fig. 5H, Additional file 1: Fig. S4B). Taken together, these data demonstrated that stimulation of adiponectin receptor signaling via its specific agonist, AdipoRon, could not only inhibit proliferation and but also induce cell death in NPC cells.

**Fig. 6:**
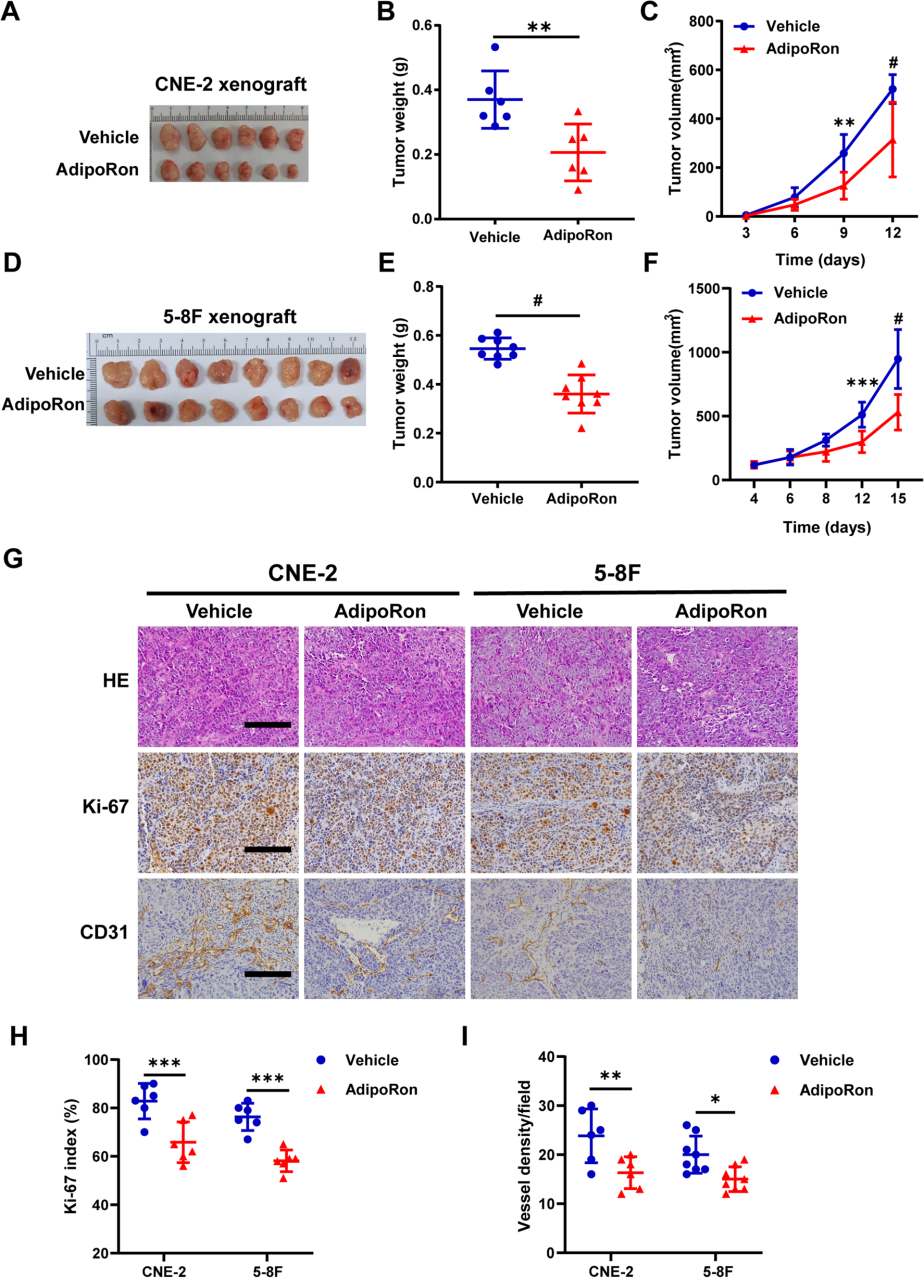
AdipoRon inhibits subcutaneous NPC-xenograft growth. **A**, **D** The tumor-bearing mice were euthanized at the end of the experiment and the subcutaneous NPC tumors were dissected and photographed. **B**, **E** Average tumor weight of CNE-2 (*n* = 6 per group) and 5-8F (*n* = 8 per group) derived xenograft tumors at the end of treatment with vehicle or AdipoRon. **C**, **F** The growth curves of the subcutaneous tumors. **G** Hematoxylin–eosin and IHC staining of Ki-67, and CD31 in the subcutaneous tumors derived from CNE-2 and 5-8F cells following the treatment with vehicle or AdipoRon. Bars: 100 μm. Quantification of Ki-67 positive cells (**H**) and vessel density (**I**) in the collected tumor tissues (*n* = 6 per group). Results are presented as mean ± SD of three independent experiments performed in triplicate. *P < 0.05, **P < 0.01, ***P < 0.001, ^#^*P* < 0.0001

The observations described above led us to investigate tumor-suppressive effects of AdipoRon in animal models. To this end, we evaluated the growth of subcutaneously implanted CNE-2- and 5-8F-dervied tumor in the nude mice. From day 4 after inoculation, the mice were orally administered 50 mg/kg AdipoRon every other day. Following the delivery, we observed a significant retardation of tumor growth (Fig. 6A–F, Additional file 1: Fig. S5A, 5C) without body weight loss (Additional file 1: Fig. S5B, 5D). Ki-67 staining of the sections prepared from AdipoRon-treated tumors revealed a sharp reduction of tumor cell proliferation in the treated vs. the untreated tumors (Fig. 6G, H). In addition, we noticed a significant decrease of vessel density, suggesting either indirect or direct inhibition of angiogenesis by AdipoRon (Fig. 6G, I). Collectively, these findings suggest that boosting adiponectin activity through oral administration of AdipoRon exhibited a strong suppressive effect against human NPC cells derived xenograft tumors.

## Discussion

The findings derived from the retrospective and prospective case–control studies established for the first time the inverse relationship between adiponectin and the risk of NPC. This newly identified inverse relationship is completely independent of other well known risk factors, such as age, EBV infection status, family history, suggesting an independent regulation of NPC development by an adipocyte-derived metabolic hormone. The current study did not stratify the correlation against body weight owing to the lack of such data in both cohorts. However, previous studies have largely ruled out the association of body weight with risk of NPC [8–10]. Thus, we do not believe that this issue will change the outcomes of this analysis. Interestingly, we did not find evidence of an association among women, which might be due to the relatively few female NPC cases. Extending investigation is required to confirm our findings, and to better elucidate sex hormones affect the relationship between adiponectin and NPC.

In corroborating the outcomes of such strong clinical associations, we further established the causative effects as well as the underlying mechanisms, of adiponectin on human NPC development. Co-incubation with adiponectin or adiponectin-receptor agonist suppressed the growth of human NPC cells, arrested cell cycle via AdipoR1- and AdipoR2-mediated AMPK activation. Importantly, adiponectin-deficiency significantly accelerated, while administration of adiponectin-receptor agonist inhibited, the growth of human NPC cell-derived xenografts in the nude mice. Taken together, these results unequivocally solidified that adiponectin is not just a correlative circulating factor but also a direct regulatory factor in the development of NPC.

Initially discovered as a crucial regulator of inflammation, energy balance, glucose/lipid metabolism [15, 18], adiponectin has been reported to have direct anti-proliferative effects in several malignant cell lines [36]. In this study, we demonstrated that adiponectin could directly suppress the growth of NPC cells by arresting cell cycles at the G0/G1 phase through regulating the expression of several cell cycle key regulators, with the activation of AMPKα as the most likely initiating signaling event. Tumor suppressor LKB1 as the critical upstream kinase, its phosphorylation leads to the activation of AMPK [35]. We found adiponectin treatment did not affect the level of total LKB1, it significantly increased the level of p-LKB1, further demonstrating that adiponectin suppresses NPC growth through activating LKB1/AMPK signaling. Furthermore, adiponectin induced AMPK activation probably suppressed the phosphorylation of ERK1/2, further enhancing the anti-proliferative effect. Recent studies have shown that AMPK signaling can play a critical role in the regulation of cancer cell proliferation via induction of apoptosis and cell cycle arrest [37–40]. Several tumor suppressor genes, such as p53, mTOR, and p27, are considered as the downstream signaling components of AMPK activation [41, 42]. 5-Aminoimidazole-4-carboxamide ribonucleoside (AICAR), a pharmacological activator of AMPK, suppresses cell growth of head and neck squamous cell carcinoma [43]. Moreover, metformin has recently received attention as an anti-tumor drug, since it induces inhibition of cancer cell proliferation via activation of AMPK signaling [44]. Our previous study has already shown that adiponectin could arrests endometrial cancer cells at the G0/G1 stage, possibly by activating AMPK [24]. Such result is well corroborated by the observation that the inhibitory effect of adiponectin on NPC cell proliferation was neutralized by inhibition of AMPK activity with a specific AMPK inhibitor, ComC, particularly adiponectin-controlled cell cycle progression in NPC cells. Besides AMPK, other molecular mechanisms could also play critical. For instance, chronic inflammation is an important even in propelling the development of NPC [3]. Owing to its well-established anti-inflammatory function, adiponectin can also prevent NPC development by suppressing proinflammatory cytokines such as IL6, tumor necrosis factor-α, and interferon γ [45], as well as inducing the expression of anti-inflammatory cytokines such as IL-10 and IL-1RA [46].

From a translational perspective, we have tested if stimulation of adiponectin receptor activity would attenuate the growth of human nasopharyngeal carcinoma, and applied AdipoRon, the first oral adiponectin receptor agonist capable of binding and activating both AdipoR1 and AdipoR2 [47], in the human NPC model. AdipoRon has emerged as a possible candidate for the treatment of different pathological conditions, including metabolic, cardiovascular, and cognitive dysfunction of Alzheimer’s disease, specifically comorbidity between depression and obesity [47–50]. In this study, we have demonstrated that oral administration of AdipoRon exhibited a robust anti-cancer effect against human NPC derived xenograft tumors, and the dosing of AdipoRon applied in our study (at 50 mg/kg) closely matched those reported in other mouse models [47, 49, 51, 52]. With multitudes of mechanisms, AdipoRon may represent a therapeutic agent that can be applied towards the treatment of human NPC.

## Conclusions

In conclusion, our findings from this study shed some new light on the pathogenesis of NPC, highlighting the importance of an adipocyte-derived endocrine hormone, adiponectin, as a crucial inhibitor to NPC tumorigenesis via AMPK activation. Further investigations are needed to establish the linkages between other adipocyte-derived endocrine hormones, in addition to adiponectin, with the progression and pathological features of NPC, such as tumor grade, vascular invasion, and metastasis. Our findings herein may provide knowledge of adiponectin as a novel therapeutic target in NPC therapy.

## Supplementary Information

**Additional file 1.** Additional file 1 provides supplementary materials, methods, figures (Fig. S1 to Fig.S5), tables (Table S1 to Table S3), and Western blots original data.

## Data Availability

The datasets used and/or analyzed during the current study are available from the corresponding author on reasonable request.

## Abbreviations

NPC: Nasopharyngeal carcinoma
EBV: Epstein-Barr virus
mRNA: Messenger RNA
HNSC: Head and neck squamous cell carcinoma
TCGA: The Cancer Genome Atlas
GEO: Gene Expression Omnibus (GEO)
CCK-8: Cell counting kit-8
siRNA: Small interfering RNA
ComC: Compound C
AdipoRs: Adiponectin receptors
AICAR: Aminoimidazole-4-carboxamide ribonucleoside
