# Supplementary material for: Adiponectin suppresses tumor growth of nasopharyngeal carcinoma through activating AMPK signaling pathway
Source: J Transl Med. 2022 Feb 14;20:89. doi: 10.1186/s12967-022-03283-0 (PMC8843017; doi:10.1186/s12967-022-03283-0)

Supplementary Materials for

**Adiponectin suppresses tumor growth of nasopharyngeal carcinoma through activating AMPK signaling pathway**

Zongmeng Zhang^1*^, Jinlin Du^2*^, Hui Shi^3^, Shuai Wang^1^, Yunjing Yan^1^, Qihua Xu^1^, Sujin Zhou^1^, Zhenggang Zhao^1^, Yunping Mu^1^, Chaonan Qian^4^, Allan Zijian Zhao^1#^, Sumei Cao^5#^ and Fanghong Li^1#^

^1^The School of Biomedical and Pharmaceutical Sciences, Guangdong University of Technology, Guangzhou 510006, China;

^2^ Department of Epidemiology and Health Statistics, School of Public Health, Guangdong Medical University, Dongguan 523808, China;

^3^ Department of Pathology, Jiangsu Province Hospital of Chinese Medicine, Affiliated Hospital of Nanjing University of Chinese Medicine, Nanjing 210029, China;

^4^ State Key Laboratory of Oncology in South China, Collaborative Innovation Center for Cancer Medicine, Sun Yat-sen University Cancer Center, Guangzhou 510060, China;

^5^ Department of Cancer Prevention Research, Sun Yat‐sen University Cancer Center, Guangzhou 510060, China;

* These authors contributed equally to this work.

**^#^ Correspondence to**:

Fanghong Li, M.D., The School of Biomedical and Pharmaceutical Sciences, Guangdong University of Technology; No.100 Waihuanxi Road, Guangzhou Higher Education Mega Center, Guangzhou 510006, Guangdong, P. R. China (Tel: +86-18027330698, E-mail: fli@gdut.edu.cn)

Or: Sumei Cao, Ph.D., Department of Cancer Prevention Research, Sun Yat-sen University Cancer Center; 651 Dongfeng Road East, Guangzhou 510060, Guangdong, P. R. China (Tel: +86-13826270146, E-mail: caosm@sysucc.org.cn).

Or: Allan Zijian Zhao, Ph.D., The School of Biomedical and Pharmaceutical Sciences, Guangdong University of Technology; No.100 Waihuanxi Road, Guangzhou Higher Education Mega Center, Guangzhou 510006, Guangdong, P. R. China (Tel: +86-18027330798, E-mail: azzhao@gdut.edu.cn)

**This file includes:**

Supplementary Materials and Methods

Fig S1 to S5

Table S1 to S3

Western blots original data

**Supplementary Materials and Methods**

**Study designs and participants**

In the hospital-based case-control study, cases (*n* = 132) and controls (*n* = 152) were consecutively collected from the serum bank of Sun Yat-sen University Cancer Center (SYSUCC) (Guangzhou, Guangdong, China) from 2009 to 2015. The patients were selected based on the following criteria: (i) Cantonese NPC patients with histologically proven NPC who had not undergone any treatment; (ii) aged 30–69 years old; (iii) lacked any severe inflammation, immune system disease, or diabetes; and (iv) had serum samples collected before treatment. Healthy controls undergoing routine health with no history of cancer were individually matched to cases based on cohort of origin, birth year, sex, date of blood collection [1]. All diagnoses of NPC were confirmed by biopsy.

In nested case-control study, 71 patients with NPC via nasopharyngeal fiberoptic examination were selected from community-based NPC screening cohort [2]. In addition to meeting the inclusion and exclusion criteria, case subjects were required to meet the following criteria: (i) maintenance of at least 100 μL of serum samples taken at time of enrollment; (ii) complete age and gender baseline data; and (iii) no serious immunodeficiency disease at time of enrollment. According to the age of the case group (±1-year), gender and blood draw time, the case control ratio was 1:2, and therefore 142 patients without NPC were randomly assigned as the control group in the cohort. All subjects signed on written informed consent, and human subject approval was obtained from the Institutional Review Board of SYSUCC (No. YP2009051).

**Blood collection and detection**

Blood samples were collected in inert separation gel coagulation tube after overnight fasting. After centrifugation at 2000g for 20 mins at 4°C, the obtained serum was separated and stored at −80°C until analysis.

The serum concentrations of EBV Epstein–Barr nuclear antigen 1–immunoglobulin A (EBNA1-IgA) (ZSGB-Bio, Beijing, China) and viral-caspid antigen–IgA (VCA-IgA) (EUROIMMUN Medizinische Labordiagnostika AG, Lübeck, Germany) were measured using an enzyme-linked immunosorbent assay kit according to manufacturer’s instructions. Positivity for ELISA-based antibodies depended on the relative optical density (rOD) value, which was calculated as the ratio of the optical density value to a reference cutoff OD value tested simultaneously. The positive criteria were ≥0.7 for EBNA1‐IgA and ≥0.8 for VCA‐IgA. The risk of nasopharyngeal carcinoma was analyzed using a prior defined assessment equation (Logit *P* = −3.934 + 2.203 × VCA-IgA + 4.797 × EBNA1-IgA) [3]. If the baseline serologic results fulfilled the definition of high risk, the participants were referred for diagnostic examinations, and different screening intervals were assigned to the high-risk (*P* ≥ 0.98), medium-risk (0.98 > *P* ≥ 0.65), and low-risk (*P* < 0.65) groups.

The adiponectin concentration was measured using a Milliplex map kit by the Luminex 200TM instrument (Millipore, Billerica, MA, USA) in the Laboratory of Cancer Prevention and Control Center of Sun Yat-sen University (Guangzhou, Guangdong, China). The measurement procedure was strictly in accordance with the standard steps of the manufacturer's kit, and the case and control serum samples were randomly distributed to each plate to ensure that the tester blindly detects the case-control state of the test sample. The coefficients of variation (CVs) and intraclass correlation coefficients (ICCs) of quality controls provided by the manufacturer were computed to evaluate the reproducibility of assays.

**Epidemiological analysis**

Descriptive characteristics of NPC cases and controls are presented as proportions or as mean ± SD. Chi-square (χ^2^) tests and independent-samples t tests were used to compare differences between cases and controls groups. Median levels of adiponectin among cases and controls were compared using the Wilcoxon rank-sum test to compare differences between groups. Adiponectin levels were divided into three groups according to the tertile of the adiponectin concentration in the control group. Odds ratios (ORs) and 95% confidence intervals (95% CIs) for risk of NPC were computed using conditional logistic regression models in prospective case-control study and unconditional logistic regression models in the hospital-based case-control study. In multivariable models, we adjusted for established or suspected risk factors of NPC, including age, sex, EBV infection, family history of NPC and smoking. Analyses stratified by sex categories using unconditional logistic regression models adjusted for matching factors. Statistical analysis was performed using SAS statistical software version 9.4 (SAS Institute, Cary, NC, USA). All hypothesis tests were two-sided test, *P* < 0.05 was considered statistically significant.

**Reference**

1. Yang M-J, Guo J, Ye Y-F, Chen S-H, Peng L-X, Lin C-Y, et al. (2018) Decreased macrophage inflammatory protein (MIP)-1α and MIP-1β increase the risk of developing nasopharyngeal carcinoma. Cancer Commun 38, 7 doi: 10.1186/s40880-018-0279-y.

2. Liu Z, Ji M-F, Huang Q-H, Fang F, Liu Q, Jia W-H, et al. Two Epstein-Barr virus-related serologic antibody tests in nasopharyngeal carcinoma screening: results from the initial phase of a cluster randomized controlled trial in Southern China. Am J Epidemiol. 2013; 177: 242-250.

3. Liu Y, Huang Q, Liu W, Liu Q, Jia W, Chang E, et al. Establishment of VCA and EBNA1 IgA-based combination by enzyme-linked immunosorbent assay as preferred screening method for nasopharyngeal carcinoma: a two-stage design with a preliminary performance study and a mass screening in southern China. Int J Cancer. 2012; 131: 406-416.

**Supplementary Figures**

**
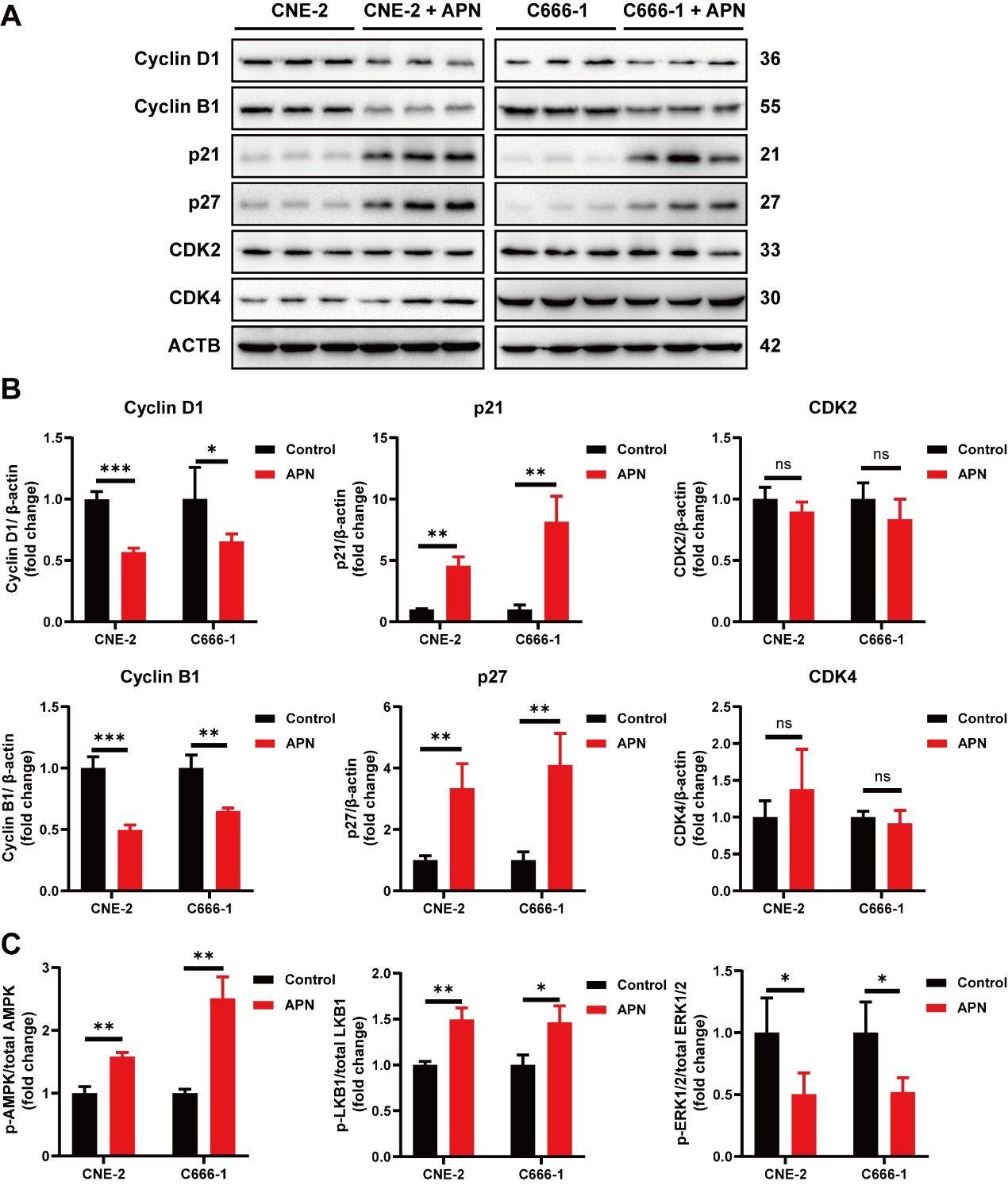
**

**Fig. S1 Adiponectin modulates cell cycle-related protein expression.**

**A** Cyclin B1, cyclin D1, p21, p27, CDK2 and CDK4 protein level was determined after the cells were exposed to adiponectin (40 μg/ml) for 48 h. **B** Quantitative analysis of cyclin B1, cyclin D1, p21, p27, CDK2 and CDK4 level was performed by densitometric analysis. **C** Quantitative analysis of p-AMPKα, p-LKB1 and p-ERK1/2 level was performed by densitometric analysis. Results are presented as mean ± SD of three independent experiments performed in triplicate. **P* < 0.05, ***P* < 0.01, ****P* < 0.001.

**
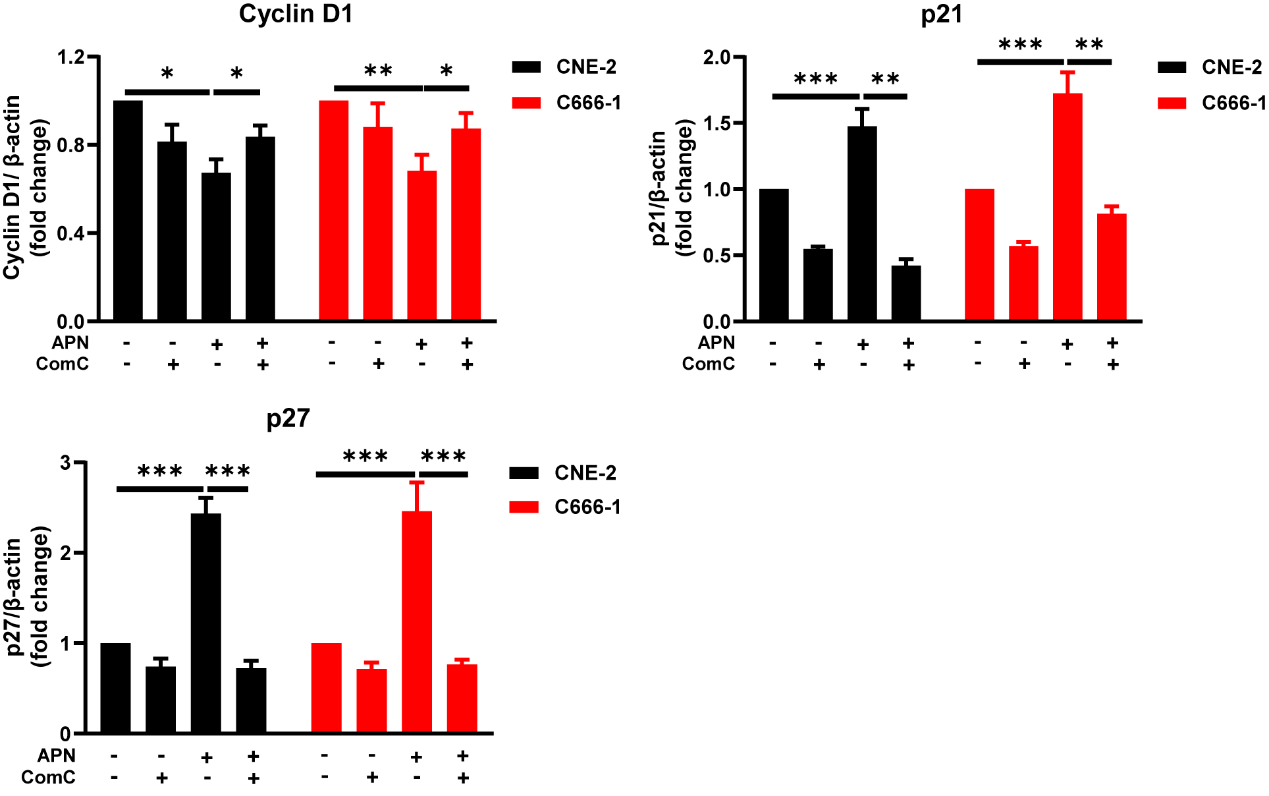
**

**Fig. S2 Quantification results of cyclin D1, p21 and p27 protein levels.**

CNE-2 and C666-1 cells were pretreated with compound C (10mM), cyclin D1, p21, and p27 protein level was then determined by Western blot analysis after the cells were exposed to adiponectin (40 μg/ml) for 48 h. Quantitative analysis of cyclin D1, p21, and p27 level was performed by densitometric analysis. Results are presented as mean ± SD of three independent experiments performed in triplicate. **P* < 0.05, ***P* < 0.01, ****P* < 0.001.


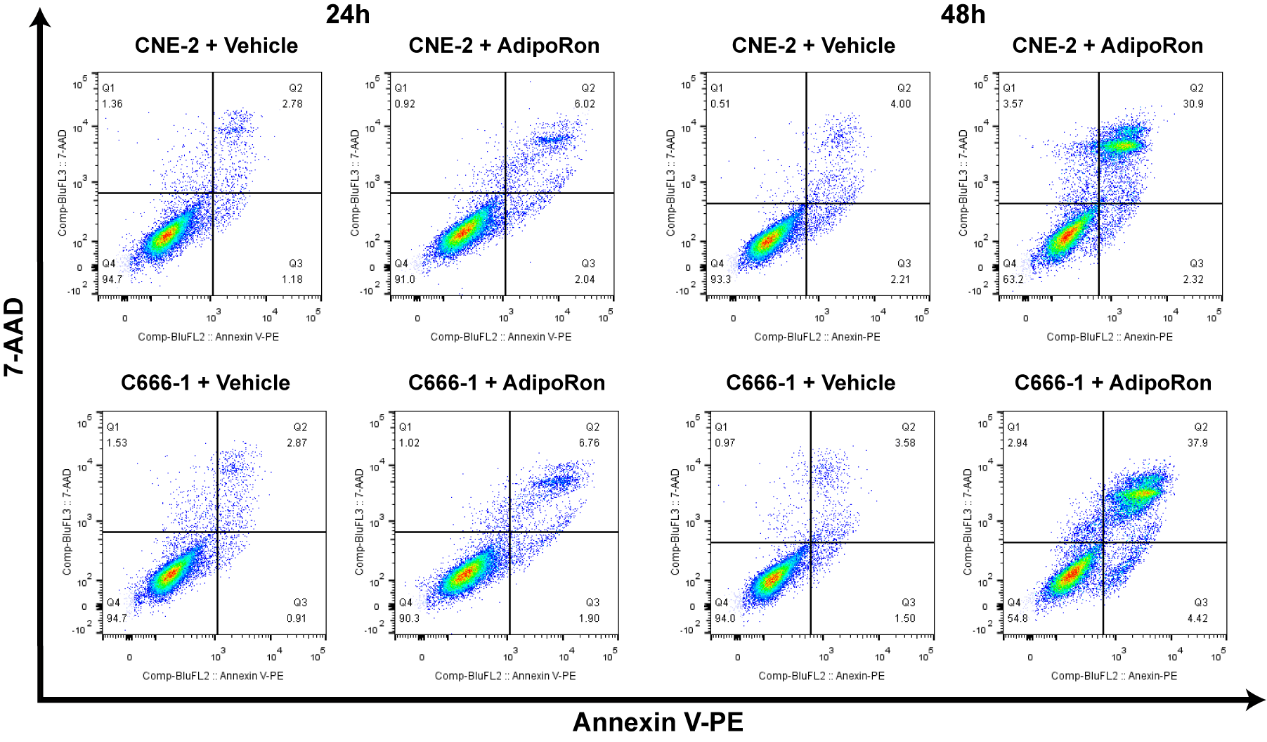


**Fig. S3 AdipoRon induces apoptosis in NPC cells.**

Annexin V/7-AAD staining of CNE2 and C666-1 cells following 24 h or 48h of exposure to 50 μM AdipoRon. Cell death was then analyzed using flow cytometer.


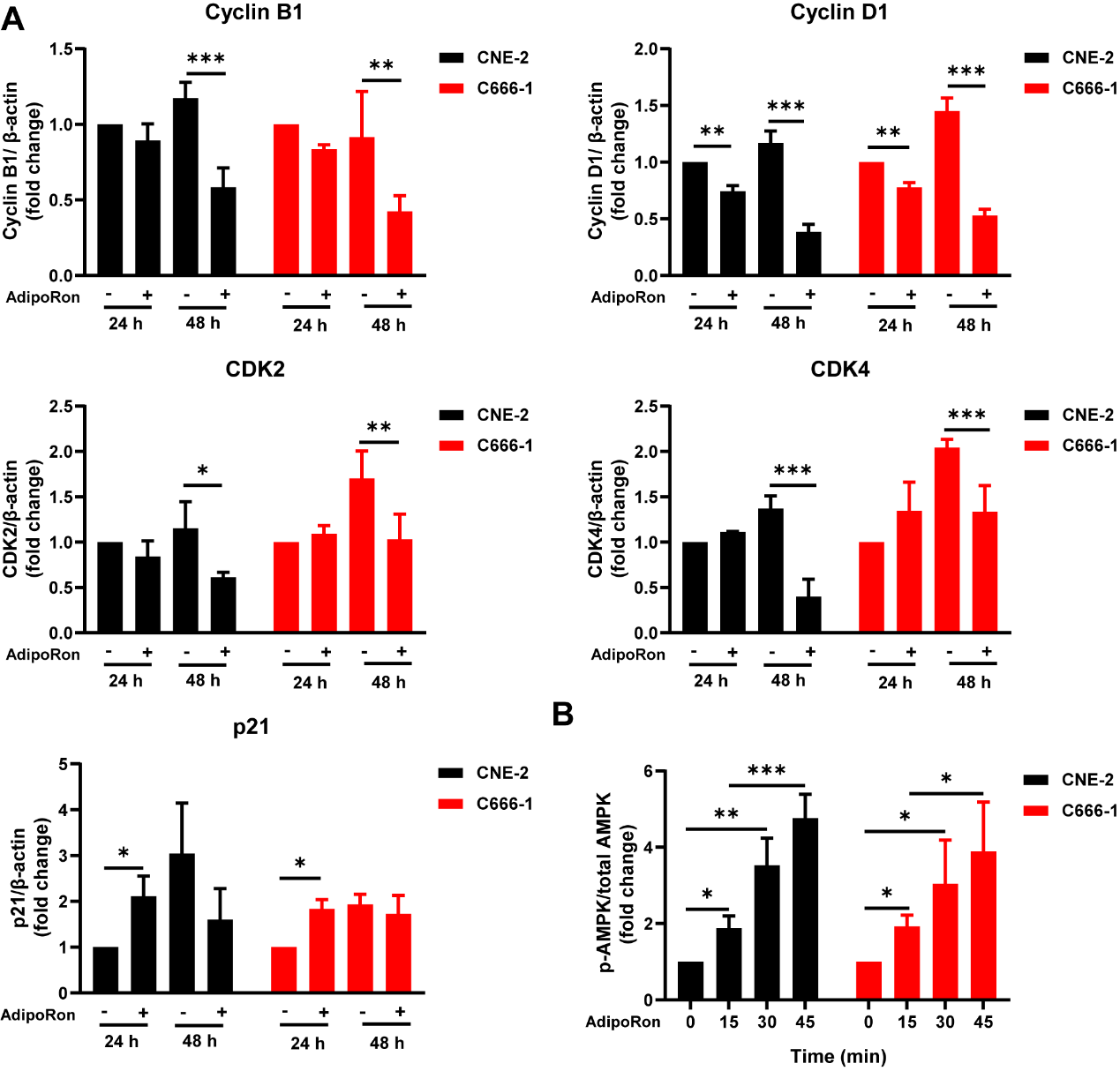


**Fig. S4 Quantification results of cyclin B1, cyclin D1, CDK2, CKD4, p21 and p-AMPKα protein levels in AdipoRon-treated CNE-2 and C666-1 cells.**

**A** The cells were treated with vehicle alone or 50 μM AdipoRon for 24h and 48h. Western blot analysis was performed to determine the cyclin B1, cyclin D1, CDK2, CKD4 and p21 protein level. Quantitative analysis was performed by densitometric analysis **B** CNE-2 and C666-1 cells were treated with 50 μM AdipoRon for the indicated time period. The p-AMPKα protein levels were determined by Western blot analysis. Quantitative analysis of p-AMPKα level was performed by densitometric analysis. Results are presented as mean ± SD of three independent experiments performed in triplicate. **P* < 0.05, ***P* < 0.01, ****P* < 0.001.


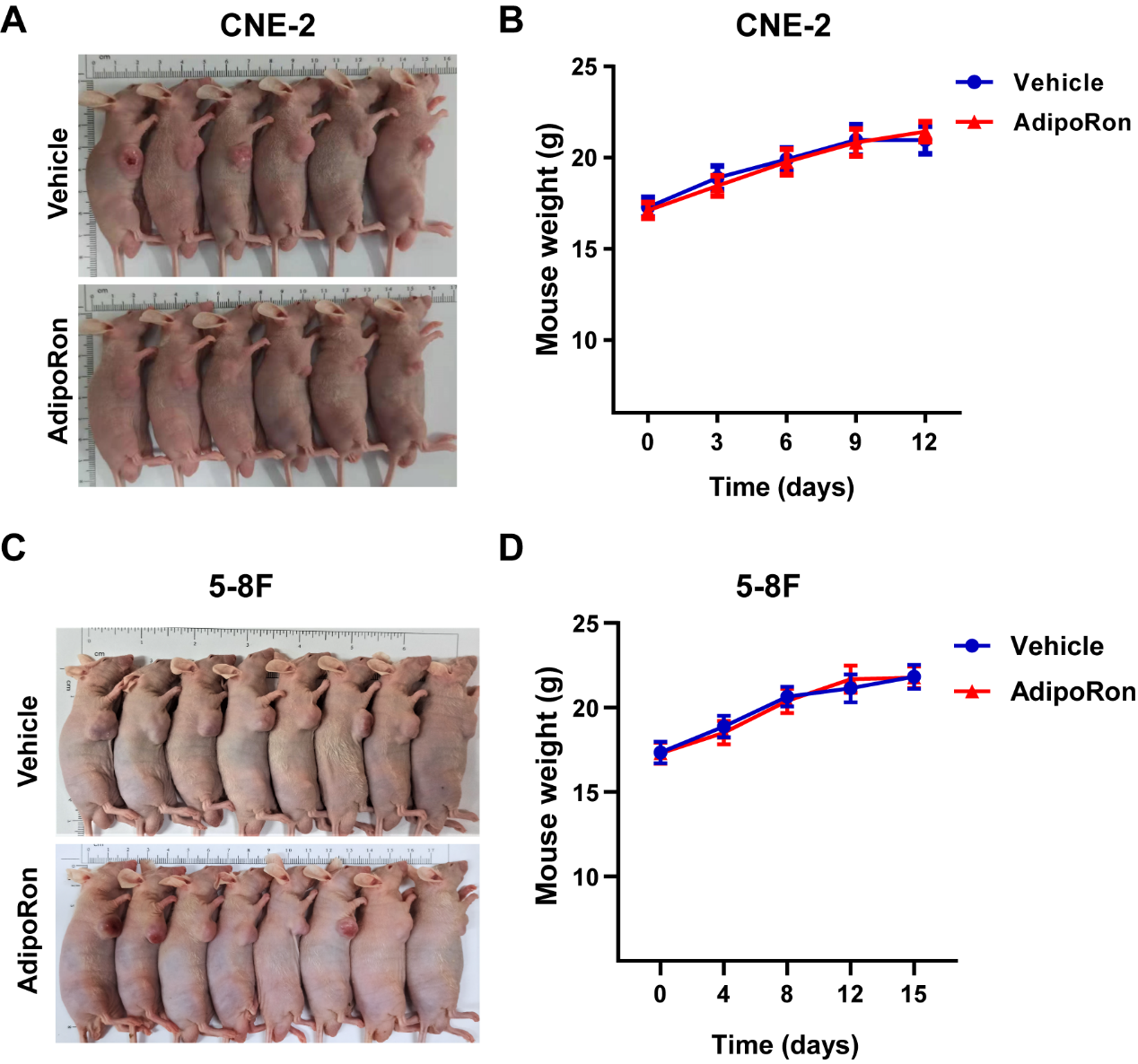


**Fig. S5 AdipoRon potently inhibits in vivo NPC tumor growth.**

**A, C** Images of subcutaneous CNE-2 cells derived tumors (*n* = 6 per group) and 5-8F cells derived tumors (*n* = 8 per group) following the treatment of vehicle alone or AdipoRon (50 mg/kg per day). **B, D** Body weight of nude mice bearing CNE-2 tumor (*n* = 6 per group) or C666-1 tumors (n = 8 per group).

**Supplementary Tables**

**Table S1.** **Characteristics of nasopharyngeal carcinoma cases and controls in the case–control and nested case–control studies.^a^**

|  | Hospital-based case-control study | | | Nested case-control study | | |
| --- | --- | --- | --- | --- | --- | --- |
| Characteristic | Cases | Controls | *P^e^* | Cases | Controls | *P*^f^ |
| Total | 132 (100) | 152 (100) |  | 71 (100) | 142 (100) |  |
| Gender |  |  | 0.531 |  |  | 1.000 |
| Male | 114 (86.4) | 135 (88.8) |  | 45 (63.4) | 90 (63.4) |  |
| Female | 18 (13.6) | 17 (11.2) |  | 26 (36.6) | 52 (36.6) |  |
| Age | 46.7 ± 9.4 | 46.0 ± 8.5 | 0.670 | 48.27 ± 8.6 | 48.27 ± 8.6 | 0.994 |
| Age group |  |  | 0.004 |  |  | 1.000 |
| 30-39 | 33 (25.0) | 37 (24.3) |  | 15 (21.1) | 30 (21.1) |  |
| 40-49 | 55 (41.7) | 61 (40.1) |  | 19 (26.8) | 38 (26.8) |  |
| 50-59 | 25 (18.9) | 48 (31.6) |  | 31 (43.7) | 62 (43.7) |  |
| 60-69 | 19 (14.4) | 6 (4.0) |  | 6 (8.4) | 12 (8.4) |  |
| VCA-IgA^b^ |  |  | <0.001 |  |  | <0.001 |
| Seronegative | 15 (11.4) | 41 (48.7) |  | 17 (23.9) | 100 (70.4) |  |
| Seropositive | 117 (88.6) | 78 (51.3) |  | 54 (76.1) | 42 (29.6) |  |
| EBNA1/IgA^c^ |  |  | <0.001 |  |  | <0.001 |
| Seronegative | 33 (25.0) | 86 (56.6) |  | 11 (15.5) | 117 (82.4) |  |
| Seropositive | 99 (75.0) | 66 (43.4) |  | 60 (84.5) | 25 (17.6) |  |
| Combined EBV serology status |  |  | <0.001 |  |  | <0.001 |
| Low-risk | 15 (11.4) | 56 (36.8) |  | 11 (15.5) | 117 (82.4) |  |
| Moderate-risk | 16 (12.1) | 47 (30.9) |  | 8 (11.3) | 18 (12.7) |  |
| High-risk | 101 (76.5) | 49 (32.3) |  | 52 (73.2) | 7 (4.9) |  |
| NPC family history |  |  | 0.017 |  |  | 0.097 |
| No | 113 (85.6) | 143 (94.1) |  | 63 (88.7) | 136 (95.8) |  |
| Yes | 19 (14.4) | 9 (5.9) |  | 8 (11.3) | 6 (4.2) |  |
| Smoking^d^ |  |  | 0.702 |  |  | 0.843 |
| No | 69 (52.3) | 76 (50.0)- |  | 42 (60.0) | 86 (60.6) |  |
| Yes | 63 (47.7) | 76 (50.0) |  | 28 (40.0) | 56 (39.4) |  |
| Adiponection^e^ (IQR) |  |  |  |  |  |  |
| Total | 1.76 (0.92-8.02) | 2.94 (1.29-12.94) | 0.003 | 1.62 (0.77-4.92) | 2.21 (1.18-9.94) | 0.060 |
| Women | 1.93 (0.80-8.90) | 1.61 (1.08-3.46) | 0.921 | 2.56 (1.17-11.22) | 2.79 (1.32-9.16) | 0.979 |
| Men | 1.72 (0.93-8.02) | 3.50 (1.31-14.31) | 0.002 | 1.45 (0.61-2.97) | 2.09 (1.15-10.14) | 0.021 |

^a^ Data are frequency (%) unless otherwise noted. VCA-IgA: viral capsid antigen-immunoglobulin A; EBNA1/IgA: Epstein–Barr nuclear antigen 1–immunoglobulin A; EBV: Epstein-Barr virus.

^b^ EBNA1/IgA positive cut-off point: 0.7

^c^ VCA-IgA positive cut-off point: 0.8

^d^ Smoking refers to smoking one cigarette every 1-3 days during a period of 6 months.

^e^ On the basis of corrected adiponectin concentration in μg/ml were represented as median.

^f^ P values were calculated by chi-square test and Wilcoxon rank-sum test.

**Table S2. Sequences of primers used in quantitative RT-PCR.**

| Target gene | primer | primer sequence |
| --- | --- | --- |
| ACTB | F | 5’-CCTGTACGCCAACACAGTGC-3’ |
|  | R | 5’-ATACTCCTGCTTGCTGATCC-3’ |
| AdipoR1 | F | 5’-ACGTTGGAGGGTCATCCCATA-3’ |
|  | R | 5’-AAACAGCACGAAACCAAGCAG-3’ |
| AdipoR2 | F | 5’-CCCTCTCTTACAAGCCCATCA-3’ |
|  | R | 5’-GAGCCAGTCTGGTAGTACATCA-3’ |

**Table S3. Sequences of small interfering RNA used in transfection.**

| Target gene | siRNA sequences |
| --- | --- |
| AdipoR1-siRNA#1 sense | 5’-CACCGTCTATTGTCATTCA -3’ |
| AdipoR1-siRNA#2 sense | 5’- TCCCTGACTGGCTAAAGGA -3’ |
| AdipoR1-siRNA#3 sense | 5’- AGAAGGGCAAACGGGTAAT -3’ |
| AdipoR1-siRNA#1 sense | 5’- ACTGGATGGTACACGAAGA -3’ |
| AdipoR1-siRNA#2 sense | 5’- TCATTCCTACCTTGCACTA -3’ |
| AdipoR1-siRNA#3 sense | 5’- TTATATGTTTCGCCCAAAT -3’ |

**Western blots original data**


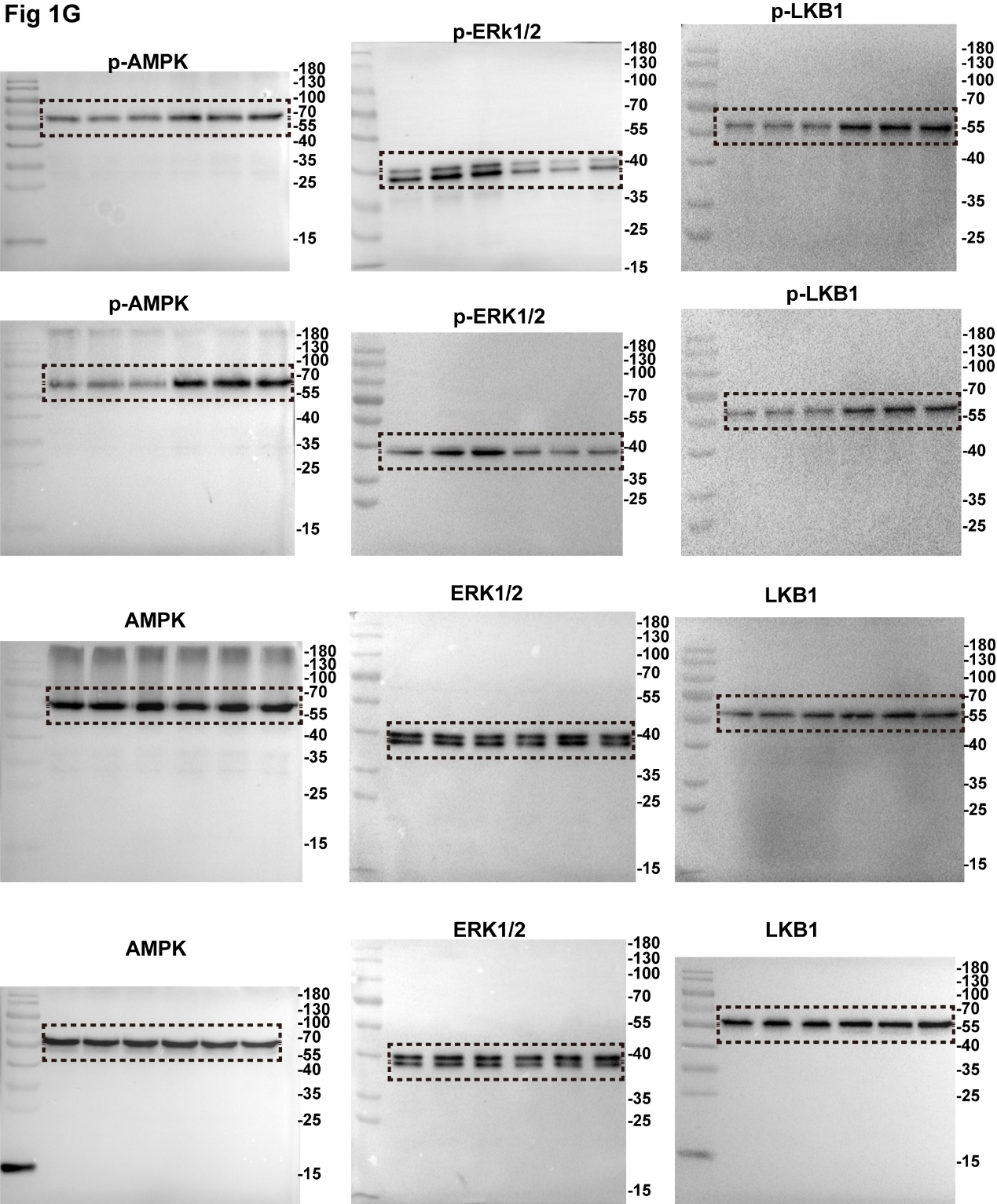


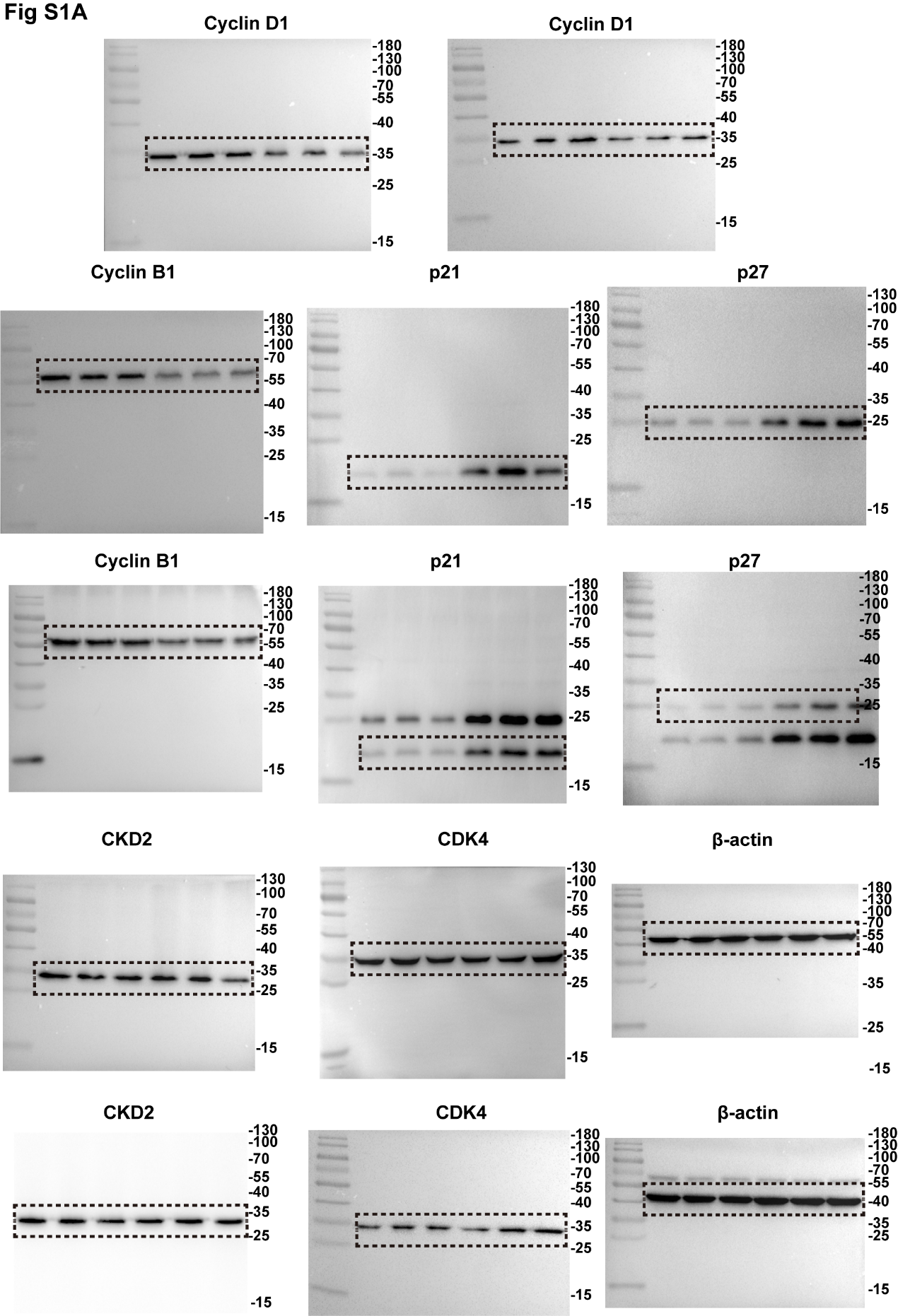

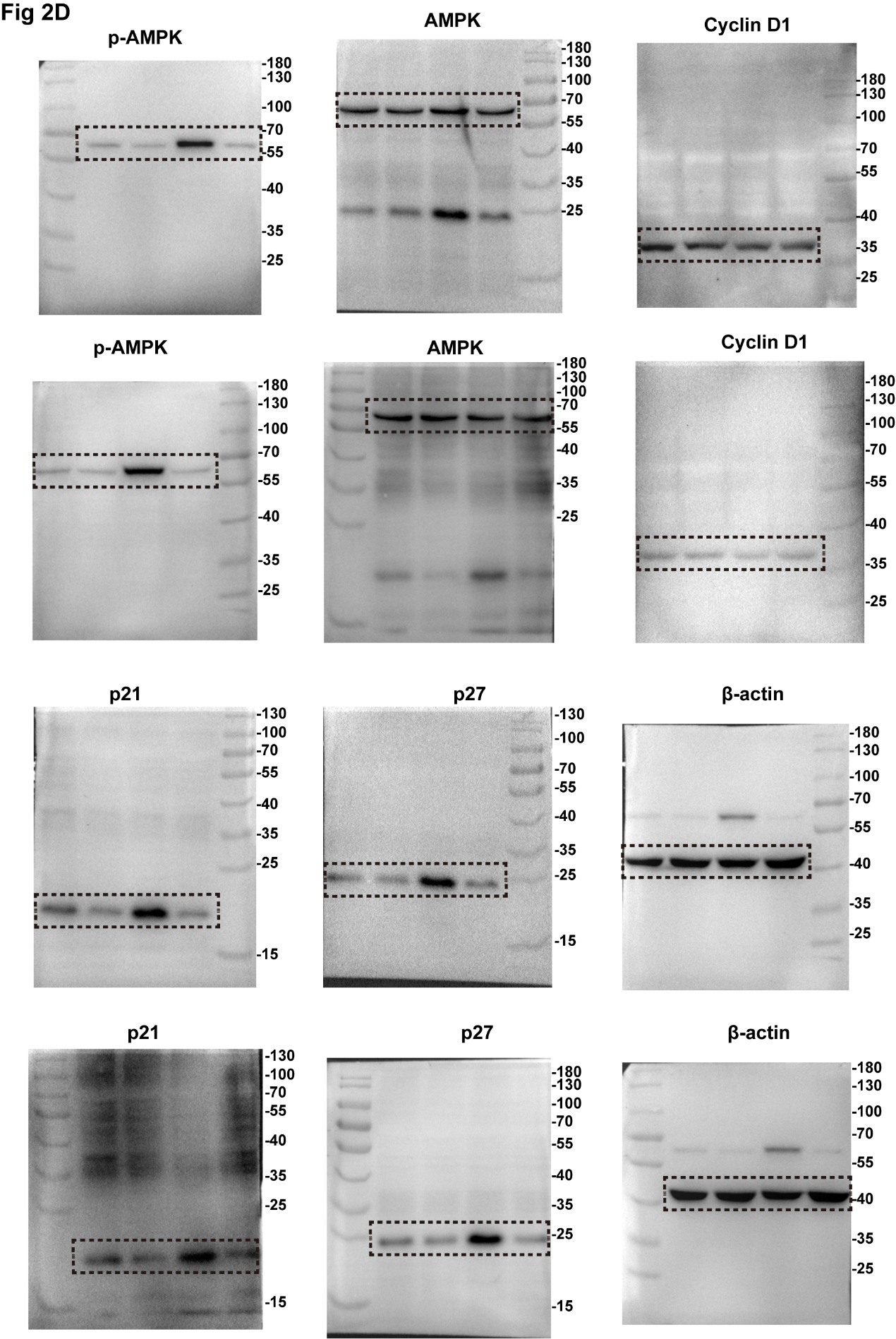

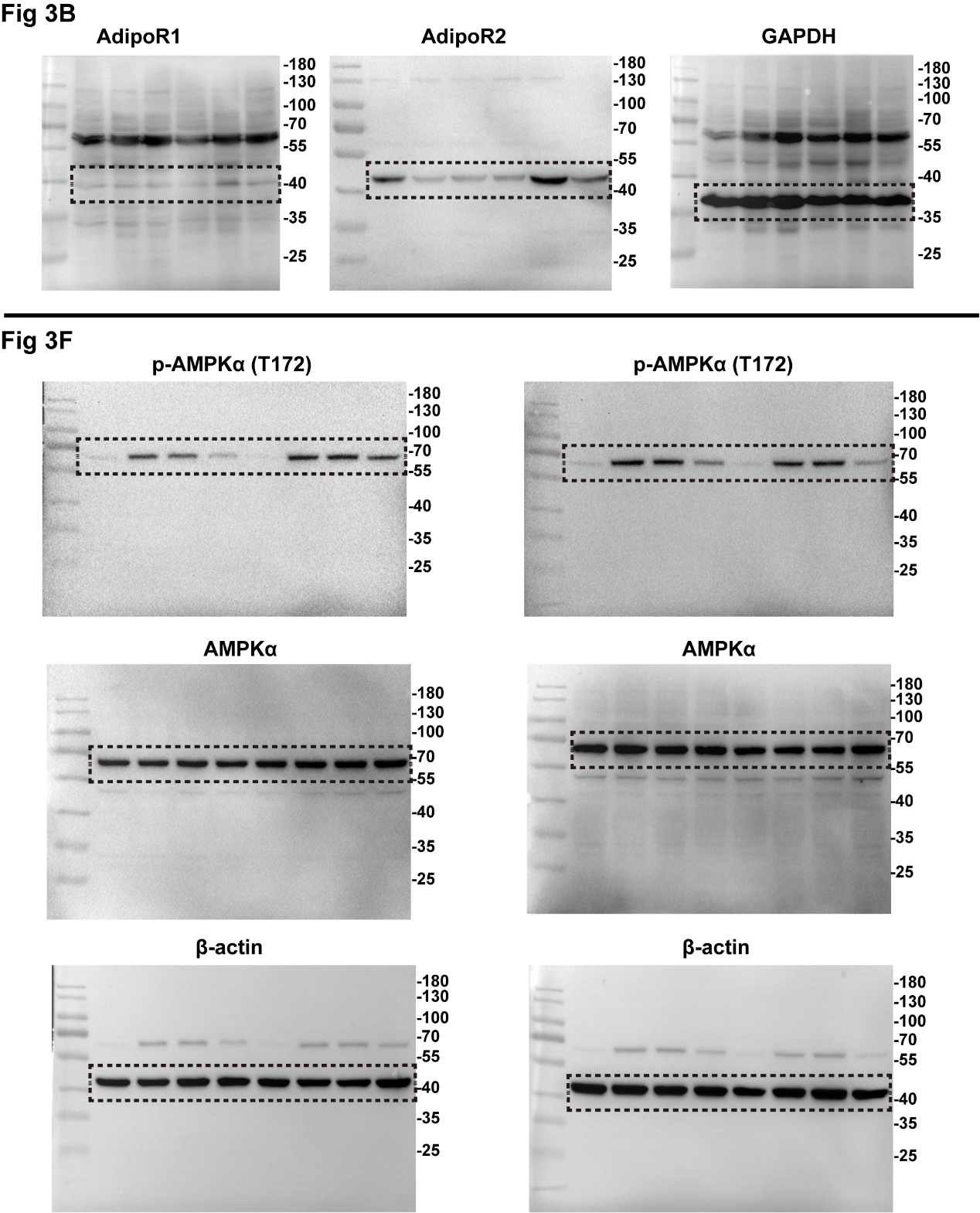

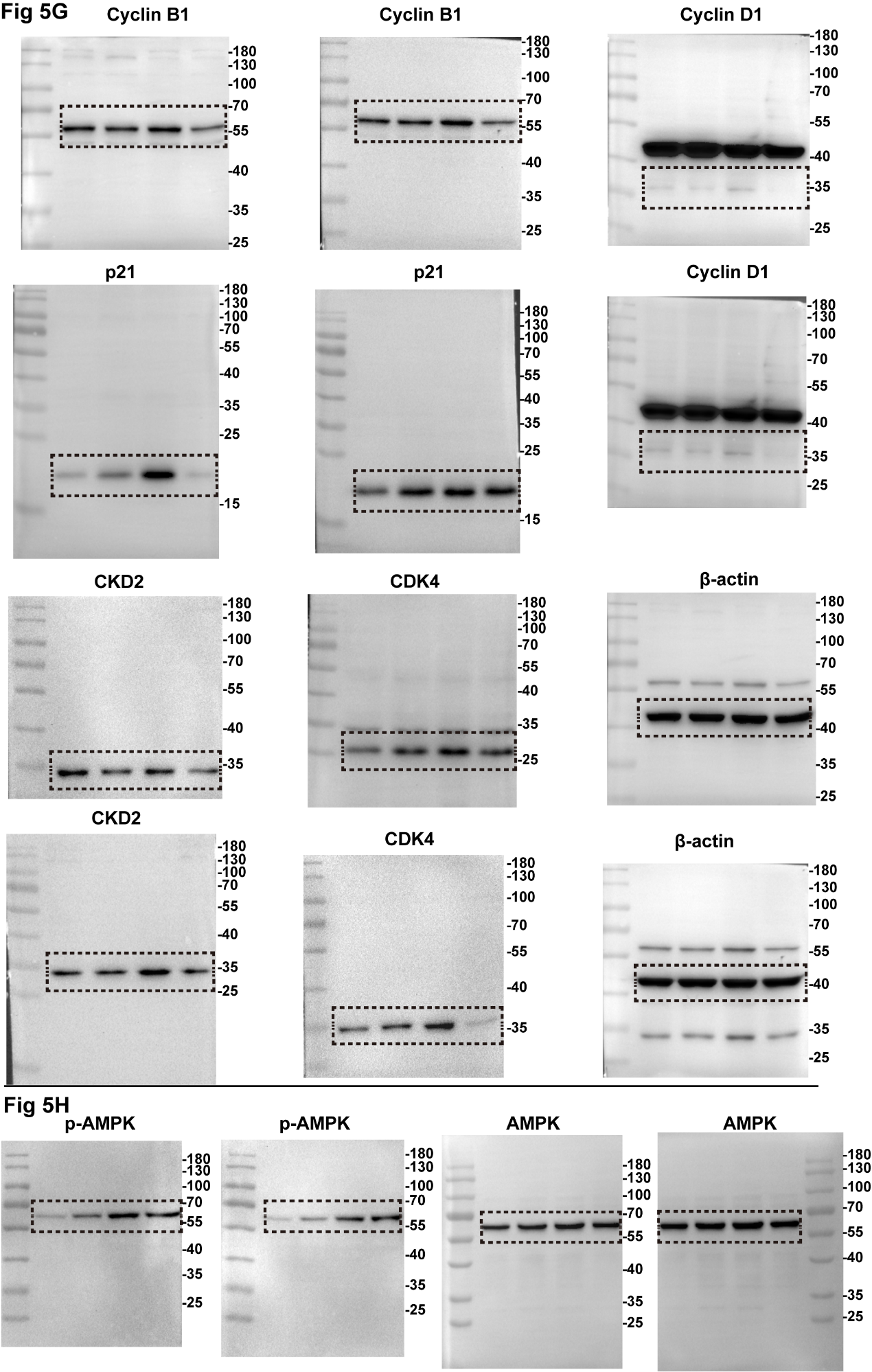

Supplement: Supplementary file 1 — Additional file 1. Additional file 1 provides supplementary materials, methods, figures (Fig. S1 to Fig.S5), tables (Table S1 to Table S3), and Western blots original data. [file 12967_2022_3283_MOESM1_ESM.docx]
